# Melatonin supplementation alleviates stocking-density stress and enhances growth, immune, and physiological performance of *Sparus aurata* cultured in groundwater-based systems

**DOI:** 10.1007/s11259-025-10992-6

**Published:** 2025-12-25

**Authors:** Ashraf. I.G. Elhetawy, Mohamed M. Abdel-Rahim, Salma Mahmoud Zeid, Omar M. El-Daly, Tamer El-Sayed Ali

**Affiliations:** 1 Aquaculture Division, National Institute of Oceanography and Fisheries, NIOF, Cairo, Egypt; 2 Oceanography Department, Faculty of Science, Alexandria University, Moharrem Bay, P.O. Box 21511, Alexandria, Egypt

**Keywords:** Melatonin, Sparus aurata, Stocking density, Physiological response, Stressful conditions, Health status

## Abstract

The intensive mariculture of *S. aurata* can promote aquaculture development, although it can also pose challenges because of predatory behavior and environmental stressors caused by intensification. The objective of this study is to alleviate stress and prevent cannibalism, thereby improving the fish’s performance and welfare. A 90-day growth experiment investigated the dietary inclusion of melatonin (ML) on *Sparus aurata* performance, physiology, immunity, histopathology, and welfare when grown in saline groundwater (36 ppt) at stocking densities (SD) of 50 fish/500 L‒tank (SD_50_) and 100 fish/500 L‒tank (SD_100_). Six fish groups in triplicate were fed three increasing levels of ML (0, 25, and 50 mg/kg^− 1^ diet) expressed as T1, T2 and T3 under SD_50_, and T4, T5, and T6 under SD_100_. Fish (*n* = 1350) weighing 16.46 ± 0.18 g/fish were grown using a flow-through system and a daily feeding rate of 4%. The results revealed significant declines in nitrogen by-product (NH_3_ and NO_2_) levels of fish fed ML in a dose density-dependent manner. Fish fed ML under both densities showed significantly improved growth indices, FCR, growth hormone, and crude protein content compared with those fed the control diet, with T2 and T6 groups achieving the highest values. ML augmentation enhanced serum lipase, protease, immunoglobulin, albumin, globulin, antioxidants, and proinflammatory cytokine interleukin-1β and lowered serum cortisol, glucose, ALT, AST, and ALP. Under both densities, the ML-fish presented healthier livers and intestines than those fed the control diet, which showed marked liver vacuoles and steatotic changes. The ML-SD interaction showed superior fish performance, particularly in groups T2 and T6; thus, it is recommended to administer dietary melatonin at 25 mg/kg for seabream reared at 50 fish per 500-liter tank and 50 mg/kg for those cultivated at 100 fish per 500-liter tank using groundwater.

**Supplementary Information:**

The online version contains supplementary material available at 10.1007/s11259-025-10992-6.

## Introduction

Aquaculture has recently played a crucial role in meeting the growing demand for animal protein globally and preserving endangered species because of the diversity of farming systems and techniques applied (Elhetawy et al. 2023). Globally, aquatic animal production in 2022 set a new record of 185 million tons (MT), a rise of 4% over that in 2020. Aquaculture produces an estimated 94.4 MT, representing 51% of the total yield (FAO 2024). In 2020, farmed gilthead seabream (*Sparus aurata*) contributed 282.1 thousand tons, accounting for 3.4% of global aquaculture production (FAO 2022). Furthermore, in 2022, the share of aquacultured *S. aurata* represented 95.8% of the global aggregate harvest, amounting to 320.630 MT, worth €1574.8 million (APROMAR 2023; Campos-Sánchez and Esteban 2024).

*Sparus aurata* is a commercially important aquaculture species in the Mediterranean, known for its high market price and suitability for aquaculture.(Lotfy et al. 2023; Mhalhel et al. 2023). More than 20 countries are involved in world production, with Turkey, Greece, and Egypt being the three leading producers, with 133,500 T, 67,000 T, and 36,000 T, respectively, of the worldwide harvest in 2022 (APROMAR 2023; Campos-Sánchez and Esteban 2024). In Egypt, *S. aurata* is the most important species utilized in mariculture, followed by meagre *Argyrosomus regius* and European seabass *Dicentrarchus labrax (*Lotfy et al. 2023), and thus could play a major role in the development of the mariculture sector if it is intensively produced in new areas employing new techniques. However, stressors such as high stocking densities, poor water quality (low oxygen, high temperature, and salinity variations), and handling have a negative impact on sea bream welfare and production. These stresses cause major economic losses for the aquaculture industry (Araújo-Luna, 2018).

The application of intensive mariculture of key important species such as *S. aurata* can support the development of aquaculture, especially when groundwater (unusable for humans and animals) is used, helping to overcome the serious impacts of freshwater scarcity on aquaculture sustainability in many regions (Abdel-Rahim et al. 2024b; El-Dahhar et al. 2024). However, S. aurata exhibits predatory behavior when subjected to stress; hence, intensifying this carnivorous species under groundwater conditions might pose serious challenges, as it limits movement and growth while increasing cannibalism. Moreover, environmental stresses, including poor water quality, negatively affect the immune system and nutrient absorption, thereby increasing vulnerability to disease. According to Acharyya et al. (2021), the two stressors mentioned earlier have a significant impact on sustainable aquaculture and are likely to surface in intensive farming conditions. In this regard, several previous studies have examined the inclusion of various natural supplements, such as essential oils and melatonin (ML), as pacifying additives in the diets of marine animals to alleviate the influences of environmental stressors and control fish cannibalism, especially at high densities, thus improving fish performance and welfare (Oyarzún-Salazar et al. 2022; Abdel-Rahim et al. 2024a; Lv et al. 2024; Ye et al. 2024).

There are two sources of melatonin, a hormone, regulates the fish’s circadian rhythm and growth. It is produced in the pineal gland and increases at night to help with sleep-wake cycles. Exogenous dietary melatonin, added to feed, enhances growth and immunity by acting as an antioxidant and immunomodulator. Both substances influence physiological processes.

ML (N-acetyl-5-methoxytryptamine) is acquiring momentum as a multipotent physiological nominee engaged in regulating and maintaining fish health (Acharyya et al. 2021). There are two sources of melatonin: endogenous and exogenous. Endogenous melatonin is a hormone produced in the pineal gland that regulates the circadian rhythm of fish (Yang et al. 2020; Veisi et al. 2021), whereas exogenous dietary melatonin is incorporated in feed and serves as a supplementary agent that can be absorbed and disseminated throughout the body to provide specific physiological advantages, such as improved growth and immunity, by functioning as an antioxidant and immunomodulator (Acharyya et al. 2021). ML is of fundamental importance to the well-being and welfare of animals at all life stages (Ye et al. 2024) and regulates the central nervous, immune, reproductive, and digestive systems (Sánchez-Vázquez et al. 2019; Yang et al. 2020). The impact of ML on fish growth is evident due to its capacity to alter the physiology of the digestive tract, and numerous indications suggest that ML influences fish intestinal microbiota in addition to increasing intestinal digestive enzyme activity (Yin et al. 2020; Zhang et al. 2021; Lv et al. 2024). Specific environmental signals may induce ML secretion by the pineal gland because of its important role in regulating various physiological roles, such as antioxidant defense (Acharyya et al. 2021). ML may directly participate in antioxidant defense mechanisms by eliminating reactive oxygen (RO) and regulating oxidative harm in fish and indirectly by increasing gene expression and ultimately activating genes encoding key antioxidant enzymes (Hardeland et al. 2003; Veisi et al. 2021). In fish, ML synthesis is understood to be mediated by external stressors and the ML response to chronic stress, thus enhancing stress resistance under environmental stress (Conde-Sieira et al. 2014; Gesto et al. 2016; Acharyya et al. 2021).

In aquatic animals, previous studies have documented the role of dietary ML in improving growth performance, the immune system, antioxidant defense, and inner organ histopathology (Li et al. 2023; Yang et al. 2023; Lv et al. 2024; Ye et al. 2024), as well as reproductive function (Aripin et al. 2015). Additionally, dietary ML was able to mitigate the harmful effects of silver nanoparticles on Nile tilapia *Oreochromis niloticus* (Veisi et al. 2021). Similarly, oral ML treatment alleviated most of the effects of high stocking density (SD) stress on rainbow trout, improved liver function and some digestive enzymes, and increased serotonin and dopamine metabolism in the hypothalamus (Conde-Sieira et al. 2014). In the same vein, the addition of ML to the rearing water of *Solea senegalensis* under acute stress of high SD enhanced the stress response of fish (Gesto et al. 2016). Also, giving ML as an intramuscular implant to Prussian carp *Carassius gibelio* for seven to thirteen weeks increased their antioxidant defense and lowered the buildup of cadmium, copper, zinc, and iron in their hepatopancreas after they were exposed to 0.4 or 4.0 mg/L of cadmium in water (Drąg-Kozak et al. 2019). On the other hand, administration of ML (25 µg/L) in the breeding water decreased the final weight and survival of Nile tilapia under normal conditions (Singh et al. 2012), while dietary ML(50 and 200 mg/kg diet) had no considerable impact on the growth performance of these fish under silver nanoparticle-induced toxicity (Veisi et al. 2021). Moreover, giving dietary ML (40 mg/kg) under laboratory culture conditions did not improve the growth performance of *S. aurata* (SD = 10 fish/m^3^) and decreased all growth indices (Amri et al. 2020). One of the most important reasons for the difference in the effect of ML on the above species is the variations in the farming conditions under which the fish were raised. In addition, SD is an important exogenous factor impacting fish growth and welfare, especially in harsh rearing conditions. In this regard, there is insufficient information on the potential impact of ML on the outgrowth phase of key marine species in the Mediterranean zone, such as *S. aurata*, especially under intensive farming. This work aims to comprehensively evaluate the impact of dietary ML supplementation on intensively cultured *S. aurata* and reveal potential improvements in groundwater quality, alleviation of stocking density stress, fish growth and related hormones (growth hormone and insulin-like growth factor 1), antioxidant status, immunity, and inner organ well-being. Apart from previous studies on melatonin supplementation, which used seawater-raised fish, this study utilized saline groundwater from wells, in an attempt to set up inland mariculture.

## Materials and methods

### Location, water source and experimental fish

This study was conducted at the El-Max Research Station, NIOF, Alexandria, Egypt. This experiment utilized groundwater whose chemical analysis is as follows: salinity 36.2 ± 0.1 ppt, pH 8.06 ± 0.1, total ammonia nitrogen (TAN) 0.41 ± 0.035 mg L^− 1^, manganese 85.2 ± 1.08 µgL^− 1^, iron 99.3 ± 2.2 µgL^− 1^, copper 5.3 ± 0.005 µg L^− 1^, zinc 6.5 ± 0.002 µg L^− 1^, cadmium 40.0 ± 1.0 µg L^− 1^, chrome 66.0 ± 2.0 µg L^− 1^, cobalt 50.0 ± 2.0 µg L^− 1^, nickel 70.0 ± 5.0 µg L^− 1^, lead 28.0 ± 3.0 µg L^− 1^, and total hardness 5823.7 ± 12.2 mg L^− 1^.

Approximately 2000 *S. aurata* fingerlings were acquired from a private fish farm and transferred to the station for acclimatization to the farming groundwater. The acclimatization phase lasted for two weeks using a 5000 L circular fiberglass tank, and the fish were fed a basal diet twice daily. Following that, a total of 1350 apparently healthy fish (mean weight 16.46 ± 0.18 g/fish) were randomly allocated into experimental fiberglass tanks for a 90-day outgrowth phase study.

### Diet formulation

A basal diet (BD) that is isonitrogenous and isolipidic was developed and supplemented with ML in varied quantities of 0, 25, and 50 mg/kg of diet (Table 1). The levels of melatonin studied were chosen based on those recommended in several prior studies (Amri et al. 2020; Chen et al. 2025; Ye et al. 2024). The feed ingredients were pulverized and blended, with a total of 300 cc of water added per kilogram of the mixture. The dough was further processed through a meat grinder, and the resultant strands were desiccated prior to being crumbled to the appropriate size. ML (tablets, each 5 mg) was acquired at a local pharmacy (https://zetapharma.net/zeta-history/dozova-melatonin-5-launch/), pulverized, and thereafter the required doses were dissolved in 1 ml of ethanol solution. The solution was then diluted with high-purity water and uniformly applied to the BD surface, which was dried for 24 h at 37 °C until a constant weight was achieved, as outlined in a prior study (Lv et al. 2024; Ye et al. 2024). The formulated diets were stored at 4 °C until use. The pellets were allowed to sit at room temperature for two hours before being fed.

**Table 1:** Formulation and proximate chemical composition (%) of a basal diet (BD) containing three levels of melatonin used in feeding gilthead sea bream Sparus aurata for a 90-day trial period

Ingredients	(g)		
	D1	D2	D3
Fish meal (65% CP- anchovy)	203	203	203
Soybean meal (45% CP)	262	262	262
Poultry by-product (56% CP)	100	100	100
Corn Gluten (62% CP)	80	80	80
Wheat meal (12% CP)	140	140	140
Yellow Corn (8% CP)	80	79.975	79.95
Spirulina (61% CP)	20	20	20
Dried Yeast (48% CP)	6	6	6
Fish oil (Herring)	60	60	60
Soyabean oil	40	40	40
Gelatin (90% CP)^1^	2	2	2
Mono calcium Phosphate (22.7% P and 16% Ca)	3	3	3
Vitamin & Mineral premix^2^	3	3	3
Vitamin C (100% purity)	1	1	1
Melatonin^3^	0	0.025	0.050
Total	1000	1000	1000
**Chemical composition**,	**(%)**		
Dry matter (DM), %	90.99	91.15	91.42
Crude protein (CP), %	40.19	40.32	40.27
Lipid, %	15.30	15.17	15.35
Fibre, %	2.23	2.20	2.26
Ash, %	6.50	6.58	6.61
NFE, %^**4**^	35.78	35.73	35.51
Gross energy, Kcal/kg DM^**5**^	5181.6	5174.6	5179.7
Gross energy, MJ/kg DM^**6**^	21.68	21.65	21.67

Diets: (D1) Basal diet (BD) without Melatonin (control ); (D2) BD supplemented with melatonin 25 mg /kg diet; (D3) BD supplemented with melatonin 50 mg /kg diet. 1 Gleatin produced by the Al-Amin Gelatin Company https://ehabelaminforgelatine.com/en; 2 Vitamin and mineral premix: Premix: vitamin A (3300 IU), vitamin D3 (410 IU), vitaminB1 (133 mg), vitamin E(150 mg), vitamin B2 (580 mg), vitamin B6 (410 mg), vitamin B12 (50 mg), biotin (9330 mg), choline chloride (4000 mg), vitamin C (2660 mg), inositol (330mg), para-amino benzoic acid (9330 mg), niacin (26.60 mg), pantothenic acid (2000 mg), manganese (325 mg), iron (200 mg), copper (25 mg), iodine, cobalt (5mg); 3 Melatonin was added at 0, 25, 50 mg/kg for treatments (1,4), (2,5), and (3,6), respectively. Melatonin was purchased from local distributor (https://zetapharma.net/zeta-history/dozovamelatonin-5-launch/); 4 Nitrogen free extract (NFE, %) = 100 - (crude protein + crude lipids + fibers + ash); 5 Gross energy was calculated based on protein, lipid, and carbohydrate values as 5.64, 9.44, and 4.11 Kcal/g, respectively; 6 Gross energy (GE), MJ/kg = (Gross energy, Kcal/ kg DM) * 0.0041868)

Note: It is worth noting that the concentration of normal melatonin levels in the basal diet has not been detected using HPLC (High-Performance Liquid Chromatography) according to (CAO, 2006 #105); therefore, it is unlikely that there will be any confounding effects on hormonal responses

### Experimental layout and rearing facilities

This experiment utilized eighteen fiberglass tanks (each 1m^3^ in volume) filled with 500 L of groundwater to culture *S. aurata* juveniles. Six treatments in triplicate (each three tanks representing one group) were administered using two stocking densities (50 and 100 fish/500 L‒tanks, water volume) and three levels of ML (0, 25, and 50 mg kg^− 1^ diet), expressed as follows:

T1 = SD_50_ML_0_, fish at a stocking density of 50 fish/tank 500 L^− 1^ were fed BD without ML.

T2 = SD_50_ML_25_, fish at a stocking density of 50 fish/tank 500 L^− 1^ were fed BD containing 25 mg kg^− 1^ ML.

T3 = SD_50_ML_50,_ fish at a stocking density of 50 fish/tank 500 L^− 1^ were fed BD containing 50 mg kg^− 1^ ML.

T4 = SD_100_ML_0_, fish at a stocking density of 100 fish/tank 500 L^− 1^ were fed BD without ML.

T5 = SD_100_ML_25_, fish at a stocking density of 100 fish/tank 500 L^− 1^ were fed BD containing 25 mg kg^− 1^ ML.

T6 = SD_100_ML_50_ fish at a stocking density of 100 fish/tank 500 L^− 1^ were fed BD containing 50 mg kg^− 1^ ML.

The initial fish stocking densities (100 fish/m³ and 200 fish/m³) in this study are based on data from previous studies (Araújo-Luna et al. 2018; Arechavala-Lopez et al. 2020; Gjije et al. 2022). This is due to the fact that the culture conditions in this study are distinct from those in the aforementioned studies, and each SD applied in these studies had its own rearing conditions. Throughout the trial period, fish were hand-fed three times daily seven days a week with a daily ration of 4% of body weight for 90 days. Since melatonin is active during the day (Chen et al. 2025), feeding times were set to be 09:30, 12:30, and 15:30. This study uses daytime feeding because implementing nighttime feeding in fish farms is difficult, and this approach aims to ensure that the study results closely align with field applications. The fish were weighed biweekly to modify the feeding rate based on variations in body weight. Fish were raised using a flow-through system under a natural light‒dark cycle. The experimental tanks were undergoing weekly cleaning and were supplied with constant aeration employing an air blower (5 h) during the study to maintain the dissolved oxygen (DO) level above > 5 mg/L.

### Water quality management

During the trial period, salinity (ppt), pH, water temperature (°C), and DO (ppm) were checked twice daily onsite at approximately the same time (10:00 a.m. and 4:00 p.m.) via a portable multimeter (Lovibond model SensoDirect 150, Germany). Weekly measurements of total ammonia nitrogen (TAN, ppm), unionized ammonia (NH_3_) and nitrite (NO_2_) contents were conducted during the study period according to APAH (2005), using a Hanna Ammonia Medium Range Portable Photometer - HI96715 (https://hanna-worldwide.com/; Hanna Instruments, Romania; https://site.jjstech.com/pdf/Hanna-Instruments/man96715_27_07_10.pdf), after collecting 0.5 L of water at a depth of 0.3 m below the water surface. NH_3_ was determined from the TAN (mg/L), salinity, pH, and temperature data.

### Growth performance and feed utilization indices

Following completion of the trial, the fish’s morphometric dimensions were estimated, their weights were recorded, and the growth performance and feeding efficiency coefficients were calculated using the following formulas (Castell et al. 1980; Saif et al. 2025):

Weight gain (WG g/fish) = final weight (FW) − initial weight (IW).

Specific growth rate (SGR, %/fish/day) = 100 × (ln FW − ln IW)/days.

Average daily gain (ADG, g) = (FW, g − IW, g)) ∕ (days).

Relative growth rate (RGR, %) = 100 × (WG/IW).

Survival (%) = 100 × (final number of fish/initial number of fish).

Feed intake (FI, g/fish/day) = feed consumption (g)/average biomass (g)×days.

Feed conversion ratio (FCR, g) = feed consumption (g)/WG (g).

Protein efficiency ratio (PER, g) = total weight gain (g)/protein intake (g).

Energy utilization (EU, %) = 100 × (energy gain (kcal)∕energy intake (kcal)).

Protein productive value (PPV, %) = 100 × (protein gain (g) ∕ protein intake (g)).

Energy gain (Kal) = Energy content in fish carcass (kcal) at the end - Energy content in fish carcass (kcal) at the start).

### Proximate chemical analyses

The whole-body composition of fish was determined using nine fish per treatment (three per replicate), which were randomly picked, weighed, and stored at − 20 °C until analysis. Measurements were conducted in accordance with the methodology established by the Association of Official Analytical Chemists (AOAC 2000). Moisture was quantified following oven drying at 105 °C until a constant weight was achieved, and ash content was assessed by incineration in a muffle furnace at 550 °C for 6 h (Nabertherm B150, Germany). A Micro-Kjeldahl machine (VELP Scientifica UDK149, Italy) was used to measure crude protein, and a Soxhlet device with petroleum ether extraction was used to measure total lipids over 16 h. The gross energy values were 16.7 kJ/g for protein, 37.4 kJ/g for lipids, and 16.7 kJ/g for carbs, respectively (NRC 2011).

### Serum biochemical variables

Following a 24-hour fasting interval, blood samples were collected from the fish at the conclusion of the experimental phase. Serum samples were collected from the caudal veins without anticoagulants of nine fish (three per replication) that had been anesthetized with 50 mg/L clove oil. After that, the serum was separated by centrifugation (3000 × g for 15 min at 4 °C) and kept at −20 °C so that it could be used for more research into biochemical parameters and antioxidant status.

#### Digestive enzymes activity

The activities of amylase, protease, and lipase were evaluated following the protocols established by Zamani et al. (2009).

#### Innate immune markers

Total protein (TP) and albumin (ALB) concentrations were measured using commercially available kits (Biodiagnostics, Giza, Egypt) (Hedayati et al. 2019), with the difference between TP and ALB quantified as globulin (GLO) (Bayunova et al. 2002). The total serum immunoglobulin (IgM) was quantified by precipitating Ig with polyethylene glycol and calculating the difference between the initial and final total protein, as per Siwicki (1993).

#### Liver enzyme activity and kidney function indicators

The technique outlined by Whitehead et al. (1991) was employed to quantify urea nitrogen, ammonia, and uric acid. Aspartate aminotransferase (AST) and alanine aminotransferase (ALT) were analyzed according to the methodology established by Bergmeyer et al. (1978) using 0.2 M DL-aspartic acid and 20 mM L-ketoglutarate as substrates and 0.2 M DL-alanine and 2 mM L-ketoglutarate, respectively. Alkaline phosphatase (ALP) was evaluated following the protocols established by Bessey et al. (1946).

#### Lipid profile

The serum triglyceride (TG) concentration was assessed using a TG quantification kit (MAK266, Sigma‒Aldrich, St. Louis, MO, USA). This assay transforms triglycerides into free fatty acids and glycerol (Fossati and Prencipe 1982). The total cholesterol (CHO) concentration is measured via free cholesterol and cholesteryl ester enzyme tests (Allain et al. 1974). Levels of high-density lipoprotein (HDL) and low-density lipoprotein (LDL) were assessed using the methodologies established by Srisawasdi et al. (2013).

#### Stress and other biochemical markers

Serum cortisol (COR) as a stress biomarker was measured according to the technique of Sadoul and Geffroy (2019). Glucose and creatinine are both important biochemical markers to evaluate shrimp health. Serum glucose (GLU) was estimated following Trinder (1969) methodology. Serum creatinine (CRE) was determined following the methods of Heinegård and Tiderström (1973).

### Antioxidative capacity

The activity status of superoxide dismutase (SOD), glutathione peroxidase (GPx), catalase (CAT), and serum malondialdehyde (MDA) levels were assessed utilizing designated kits (ZellBio GmbH, Germany) in accordance with the manufacturer’s guidelines and as previously illustrated by Hoseini and Yousefi (2019); Yousefi et al. (2019). The serum SOD concentration was evaluated by quantifying the conversion of superoxide anions into hydrogen peroxide. The serum catalase activity was evaluated by quantifying the decomposition rate of hydrogen peroxide. Serum MDA concentrations were quantified using a reaction with thiobarbituric acid at 95 °C, whereas GPx levels were assessed after 30 min of centrifugation at 4 °C.

### Growth hormone (GH), insulin-like growth factor 1 (IGF1), and interleukin 1β (IL-1β) were evaluated via ELISA kits

In the absence of seabream-specific ELISAs, fish-specific ELISA kits produced by Cusabio were utilized. GH levels in fish serum were measured following the manufacturer’s guidelines with fish-specific ELISA-GH kits (https://www.cusabio.com/uploadfile/Ins/CSB-E12121Fh.pdf*)* with sensitivity of 312.5 pg/ml, and detection wavelength of 450 nm. In addition, IGF-1 levels in fish serum were estimated using fish-specific IGF-1 ELISA kits (https://www.cusabio.com/uploadfile/Ins/CSB-E12122Fh.pdf*)* with sensitivity of 31.25 pg/ml, and detection wavelength of 450 nm. The level of the pro-inflammatory cytokine IL-1β in serum was determined employing a fish ELISA kit for interleukin 1β according to the manufacturer’s protocol (https://www.cusabio.com/uploadfile/Ins/CSB-E13259Fh.pdf*)* with sensitivity of 0.78 pg/ml and detection wavelength of 450 nm. 1 picograms (pg)/ml = 0.001 nanograms (ng)/ml.

### Histomorphometric inspection

At the end of the trial, fish (*n* = 9 specimens/group) were necropsied to obtain the livers and intestines (foregut and hindgut). The samples were subsequently fixed in a 10% neutral buffered formalin solution for 48 h. Following fixation, the tissue samples were embedded using the paraffin embedding technique outlined by Bradford (1976). The tissues were dehydrated in varying amounts of ethyl alcohol, cleaned with xylene, embedded in paraffin wax, cut into 5-µm slices, and colored with hematoxylin and eosin (H&E). A digital camera (Leica EC3, Leica, Germany) connected to a microscope (Nikon E200, Tokyo, Japan) was subsequently employed to capture many illustrative microscopy images of the prepared sections.

### Data statistics and analysis

To detect the effect of SD on *S. aurata* performance, statistical analysis was performed using one-way analysis of variance (ANOVA), while two-way MANOVA was completed to estimate the effect of ML, SD, and their interaction. Post hoc comparisons were performed using Tukey’s test at a significance level of 0.05. The approach delineated by Assaad et al. (2015) was followed in this statistical methodology. Reported outcomes are delivered as means ± standard error of the mean (SEM), providing a comprehensive definition of the main direction and variability within the dataset. The homogeneity of using the Levene’s test, and the normality tests, which include the Kolmogorov-Smirnov and Shapiro-Wilk tests were also performed using SPSS version 26. Additionally, the effect size tests were conducted with Eta-squared (η2) in SPSS (Detailed statistical analysis results are provided in the supplementary file.).

## Results

### Water quality findings

The water quality results indicated no significant variations in temperature or pH among the investigated groups; however, significant variations were detected in other water quality indicators among the treatments. The mean water temperature was 23.42 ± 0.12 °C, and the mean pH was 8.17 ± 0.008. Table 2 shows how changes in density and ML dose cause changes in DO, TAN, NH_3_, and NO_2_. The oxygen levels were significantly lower (P *< 0.05*) in tanks with higher density compared to those with lower density. Treatments containing ML showed higher DO levels than those without ML at both densities. Different groups that were given ML exhibited reduced levels of TAN, NH_3_, and NO_2_ in comparison to the control groups (P *< 0.05*). The greatest significant reductions occurred in the SD_50_ML_25_ and SD_50_ML_50_ groups, revealing an interaction effect between ML dose and SD level, as illustrated in Table 2. ML at a concentration of 50 mg/kg feed yielded better ecological effects at higher density, whereas ML at 25 mg/kg feed produced superior environmental outcomes at lower density.

**Table 2:** Water quality variables of gilthead sea bream Sparus aurata fed different levels of melatonin under different stocking densities in groundwater conditions for 90 days

SD	SD_50_			SD_100_			(*P*-value)		
ML dose	ML_0_	ML_25_	ML_50_	ML_0_	ML_25_	ML_50_			
Treatments	SD_50_ML_0_	SD_50_ML_25_	SD_50_ML_50_	SD51_00_ML_0_	SD_100_ML_25_	SD_100_ML_50_	SD	ML	SD*ML
Temperature (^o^C)	23.40 ± 0.32	23.43 ± 0.40	23.41 ± 0.31	32.42 ± 0.35	32.47 ± 0.31	23.38 ± 0.47	0.975	0.986	0.994
pH	8.18 ± 0.03	8.16 ± 0.03	8.17 ± 0.02	8.19 ± 0.02	8.16 ± 0.02	8.17 ± 0.02	0.760	0.606	0.973
DO (ppm)	6.52 ± 0.03^**abc**^	6.64 ± 0.07^**a**^	6.60 ± 0.09^**ab**^	6.31 ± 0.04^**c**^	6.37 ± 0.13^**bc**^	6.39 ± 0.02^**bc**^	0.002	0.445	0.908
TAN (ppm)	0.430 ± 0.02^**c**^	0.293 ± 0.03^**d**^	0.340 ± 0.03^**d**^	0.592 ± 0.03^**a**^	0.526 ± 0.01^**ab**^	0.499 ± 0.02^**bc**^	0.001	0.003	0.278
NH_3_ (ppb)	26.03 ± 1.68^**bc**^	17.03 ± 1.70^**d**^	20.20 ± 2.16^**cd**^	36.90 ± 3.92^**a**^	31.07 ± 1.39^**ab**^	29.47 ± 1.90^**b**^	0.001	0.013	0.583
NO_2_ (ppb)	63.58 ± 4.56^**bc**^	37.92 ± 2.54^**d**^	55.86 ± 4.17^**c**^	93.09 ± 3.11^**a**^	70.00 ± 4.04^**b**^	61.69 ± 5.91^**bc**^	0.001	0.001	0.016

Mean values in the same row with different superscript letters are significantly different (P < 0.05). Data represent mean ± SEM, n = 3 replicates

SD = stocking density, ML = melatonin. SD50ML0 = fish at stocking density of 50 fish 500L-1 fed BD without ML, SD50ML25 = fish at stocking density of 50 fish 500L-1 fed BD containing 25 mg kg-1 ML. SD50ML50 = fish at stocking density of 50 fish 500L-1 fed BD containing 50 mg kg-1 ML. SD100ML0 = fish at stocking density of 100 fish 500L-1 fed BD without ML. SD100ML50 = fish at stocking density of 100 fish 500L-1 fed BD containing 25 mg kg-1 ML. SD100ML50 = fish at stocking density of 100 fish 500L-1 fed BD containing 50 mg kg-1 ML. DO = Dissolved oxygen. TAN = total ammonia nitrogen. NH3 = unionized ammonia. NO2 = nitrite

### Growth performance and feed efficacy

Dietary supplementation of ML in the *S. aurata* diet considerably (*P* < 0.05) improved growth performance, feed effectiveness, and survival rate (Table 3) under both SD_50_ and SD_100_ conditions. There were significant improvements in final weight (FW), weight gain (WG), ADG, SGR, and RGR in fish of the ML-supplemented groups across both densities compared to the control groups (SD_50_ML_0_ and SD_100_ML_0_). Furthermore, fish fed ML-enriched diets exhibited improved feed utilization indices, as demonstrated by lowered FCR and enhanced PER, PPV, and energy utilization levels ​​compared to those fed the control diet under both densities. The interaction impact between ML and SD levels was dose-density-dependent and modified *S. aurata* performance accordingly. Under SD_50_ conditions, administering ML at a dose of 25 mg/kg diet was adequate to accomplish higher growth performance, survival rate, and feed efficiency, while with increasing SD (100 fish/500 L‒tanks), there was a need to increase the ML dose to 50 mg/kg diet to achieve better growth parameters, survival rate, and FCR. The interaction between ML at 25 mg/kg feed and SD_50_ (SD_50_ML_25_ group) demonstrated significantly higher FW, WG and ADG than all groups tested. Fish given ML at 50 mg/kg diet at SD_100_ (SD_100_ML_50_ group) displayed significantly higher growth parameters and better FCR than all groups tested, except those of the SD_50_ML_25_ group, while both groups maintained the same SGR, RGR, FCR, PER, and survival rate. Increasing the ML dose to 50 mg under SD_50_ conditions did not further boost the FW, WG, and FCR, and the impact on fish performance was comparable to that of the low dose (25 mg) under SD_100_ conditions. SD alone had a considerable influence (*P* < 0.05) and adversely affected fish performance, resulting in lower FW, WG, ADG, SGR, FCR, PER, and energy gain values ​​in the SD_100_ML_0_ group compared to the SD_50_ML_0_ group.

**Table 3:** Growth performance and feed utilization of gilthead sea bream Sparus aurata fed different levels of melatonin under different stocking densities in groundwater conditions for 90 days

SD	SD_50_			SD_100_			(*P*-value)		
ML dose	ML_0_	ML_25_	ML_50_	ML_0_	ML_25_	ML_50_			
Treatments	SD_50_ML_0_	SD_50_ML_25_	SD_50_ML_50_	SD_100_ML_0_	SD_100_ML_25_	SD_100_ML_50_	SD	ML	SD*ML
Initial weight (g/fish)	16.46 ± 0.18	16.46 ± 0.21	16.46 ± 0.11	16.48 ± 0.10	16.46 ± 0.08	16.44 ± 0.09	0.984	0.988	0.988
Final Weight (g/fish) ^**1**^	40.50 ± 0.50^**d**^	47.00 ± 0.33^**a**^	42.77 ± 0.21^**c**^	36.62 ± 0.09^**e**^	42.55 ± 0.27^**c**^	44.95 ± 0.09^**b**^	0.001	0.001	0.001
Weigh gain, (g/fish)	24.04 ± 0.57^**d**^	30.55 ± 0.54^**a**^	26.31 ± 0.20^**c**^	20.14 ± 0.14^**e**^	26.12 ± 0.35^**c**^	28.49 ± 0.10^**b**^	0.001	0.001	0.001
ADG (g/fish/day)	0.267 ± 0.01^**d**^	0.339 ± 0.01^**a**^	0.292 ± 0.00^**c**^	0.224 ± 0.00^**e**^	0.290 ± 0.00^**c**^	0.317 ± 0.00^**b**^	0.001	0.001	0.001
SGR (%/fish/day)	1.00 ± 0.02^**c**^	1.17 ± 0.02^**a**^	1.06 ± 0.01^**bc**^	0.89 ± 0.01^**d**^	1.06 ± 0.01^**bc**^	1.12 ± 0.01^**ab**^	0.001	0.001	0.001
RGR (%)	246.1 ± 5.1^**c**^	285.7 ± 5.59^**a**^	259.9 ± 1.72^**bc**^	222.2 ± 1.46^**d**^	258.9 ± 2.98^**bc**^	273.1 ± 1.24^**ab**^	0.001	0.001	0.001
Survival (%)	88.00 ± 1.15^**cd**^	97.33 ± 0.67^**a**^	92.00 ± 1.15^**bc**^	85.33 ± 0.67^**d**^	91.33 ± 0.67^**bc**^	94.00 ± 1.15^**ab**^	0.014	0.001	0.004
Feed intake (g/fish)	39.73 ± 0.79^**c**^	44.62 ± 0.60^**a**^	42.29 ± 0.53^**ab**^	36.70 ± 0.34^**d**^	41.82 ± 0.30^**bc**^	42.97 ± 0.46^**ab**^	0.002	0.001	0.007
FCR	1.653 ± 0.01^**b**^	1.457 ± 0.01^**c**^	1.610 ± 0.03^**b**^	1.820 ± 0.01^**a**^	1.603 ± 0.03^**b**^	1.507 ± 0.01^**c**^	0.001	0.001	0.001
PER (g)	1.503 ± 0.01^**b**^	1.697 ± 0.01^**a**^	1.547 ± 0.03^**b**^	1.367 ± 0.01^**c**^	1.547 ± 0.03^**b**^	1.647 ± 0.01^**a**^	0.001	0.001	0.001
PPV (%)	30.76 ± 0.80^**cd**^	40.13 ± 1.20^**a**^	33.96 ± 0.32^**bc**^	27.71 ± 0.37^**d**^	34.75 ± 0.19^**b**^	33.38 ± 0.84^**bc**^	0.001	0.001	0.018
Energy gain (Kcal)	63.09 ± 2.01^**c**^	80.19 ± 0.74^**a**^	70.36 ± 0.19^**b**^	55.70 ± 1.01^**d**^	70.94 ± 0.76^**b**^	75.42 ± 1.26^**ab**^	0.001	0.001	0.001
Energy utilization (%)	30.64 ± 0.65^**bc**^	34.75 ± 0.59^**a**^	32.13 ± 0.32^**ab**^	29.29 ± 0.50^**c**^	32.78 ± 0.33^**ab**^	33.90 ± 0.91^**a**^	0.304	0.001	0.017

Mean values in the same row with different superscript letters are significantly different (P < 0.05). Data represent mean ± SEM, n = 3 replicates

SD50ML0 = fish at stocking density of 50 fish 500L-1 fed BD without ML, SD50ML25 = fish at stocking density of 50 fish 500L-1 fed BD containing 25 mg kg-1 ML.SD50ML50 = fish at stocking density of 50 fish 500L-1 fed BD containing 50 mg kg-1 ML. SD100ML0 = fish at stocking density of 100 fish 500L-1 fed BD without ML. SD100ML50 = fish at stocking density of 100 fish 500L-1 fed BD containing 25 mg kg-1 ML. SD100ML50 = fish at stocking density of 100 fish 500L-1 fed BD containing 50 mg kg-1 ML. ADG, g/fish/day = (final body weight - initial body weight)/ number of experimental days. SGR, %/fish/day = = 100 × (ln (final body weight)-ln (initial body weight))/days. RGR = Relative growth rate %FCR = feed conversion ratio = g feed consumed/g weight gained PER, g = 100× (total weight gain (g) / protein intake (g)). PPV, % = 100 × (protein gain (g) ∕protein intake (g)). Energy gain, Kcal = (energy content in fish carcass at the end)- (energy content in fish carcass at the start). Energy utilization, % = 100 ¬Energy gain (kcal) ∕Energy intake (kcal)

### Whole-body chemical composition

Table 4 depicts the effects of the dietary ML concentration and SD on the *S. aurata* carcass composition. The inclusion of ML noticeably (*P* < 0.05) altered the whole carcass composition under both SD conditions, as indicated by increased dry matter and protein contents and reduced lipid content compared with those of the control groups. Notably, these modifications appeared as a result of a dose-dependent interaction between ML and SD. Compared with the SD_50_ML_0_ and SD_100_ML_0_ groups, the interaction between the SD and ML levels increased protein content in all ML fish, increased the dry matter content in the SD_50_ML_25_ and SD_100_ML_50_ groups, and decreased the fat content in the SD_50_ML_25_ and SD_100_ML_50_ groups. Aside from that, when ML and SD_50_ worked together, the SD_50_ML_25_ group had significantly less carcass energy than any of the other groups that were tested. The ML and SD interactions did not change the amount of ash in the fish under any of the SDs. However, these interactions greatly decreased the amount of ash in the SD_50_ML_50_ group compared to the SD_100_ML_0_ group. The fact that the SD_100_ML_0_ group’s protein content dropped significantly compared to the SD_50_ML_0_ group suggests that SD alone changed the fish’s protein content, since other carcass parameters stayed the same.

**Table 4:** Carcass composition of gilthead sea bream Sparus aurata fed different levels of melatonin under different stocking densities in groundwater conditions for 90 days

SD	SD_50_			SD_100_			(*P*-value)		
ML dose	ML_0_	ML_25_	ML_50_	ML_0_	ML_25_	ML_50_			
Treatments	SD_50_ML_0_	SD_50_ML_25_	SD_50_ML_50_	SD_100_ML_0_	SD_100_ML_25_	SD_100_ML_50_	SD	ML	SD*ML
Dry matter, %	32.97 ± 0.62^**c**^	35.40 ± 0.35^**a**^	33.99 ± 0.06^**bc**^	33.32 ± 0.39^**c**^	33.82 ± 0.27^**bc**^	34.58 ± 0.19^**ab**^	0.482	0.004	0.021
Protein, %	50.75 ± 0.19^**c**^	54.56 ± 0.77^**a**^	52.59 ± 0.24^**b**^	48.78 ± 0.19^**d**^	50.23 ± 0.32^**c**^	52.46 ± 0.24^**b**^	0.000	0.000	0.001
Ether extract, %	34.09 ± 0.16^**ab**^	29.99 ± 0.76^**d**^	33.03 ± 0.14^**bc**^	34.94 ± 0.14^**a**^	35.14 ± 0.35^**a**^	32.74 ± 0.21^**c**^	0.000	0.000	0.000
Ash, %	14.30 ± 0.44^**ab**^	14.06 ± 0.83^**ab**^	12.98 ± 0.38^**b**^	14.60 ± 0.15^**a**^	13.59 ± 0.50^**ab**^	13.88 ± 0.12^**ab**^	0.539	0.132	0.372
Carcass energy, Kcal/100gm	608.04 ± 2.28^**a**^	590.80 ± 6.71^**b**^	608.42 ± 2.69^**a**^	604.94 ± 0.98^**a**^	615.07 ± 5.10^**a**^	604.93 ± 0.72^**a**^	0.079	0.010	0.113

Mean values in the same row with different superscript letters are significantly different (P < 0.05). Data represent mean ± SEM, n = 3 replicates

SD50ML0 = fish at stocking density of 50 fish 500L-1 fed BD without ML, SD50ML25 = fish at stocking density of 50 fish 500L-1 fed BD containing 25 mg kg-1 ML. SD50ML50 = fish at stocking density of 50 fish 500L-1 fed BD containing 50 mg kg-1 ML.SD100ML0 = fish at stocking density of 100 fish 500L-1 fed BD without ML. SD100ML50 = fish at stocking density of 100 fish 500L-1 fed BD containing 25 mg kg-1 ML. SD100ML50 = fish at stocking density of 100 fish 500L-1 fed BD containing 50 mg kg-1 ML

### Haemato-biochemical findings

#### Digestive enzyme activity

Table 5 shows the digestive enzyme activity in *S. aurata* fed the control and ML-supplemented diets under varying SD conditions. The individual effects of SD and the interaction between the ML dose and SD did not affect amylase activity in any of the fish groups. Compared with those fed a control diet, the fish fed the ML-supplemented diets under both SD conditions presented higher lipase and protease activities. For lipase, there was a significant (*P* < 0.05) effect of ML supplementation, with increased activity in fish fed ML-supplemented diets, but no significant effect of SD and no significant interaction between ML and SD (P *≥ 0.05*). For protease, there were significant dose-dependent increases in activity in response to both SD and ML, but no interaction between them (P *≥ 0.05*). Seabream in the SD_50_ML_50_ group displayed the highest protease activity, whereas those in the SD_100_ML_50_, SD_50ML50,_ and SD_50_ML_25_ groups presented the highest lipase levels. The impact of SD alone was significant, as the lowest values ​​of both enzymes were recorded in the SD_100_ group (SD_100_ML_0_ group).

**Table 5:** Digestive enzyme activity in gilthead sea bream Sparus aurata fed different levels of melatonin under different stocking densities in groundwater conditions for 90 days

SD	SD_50_			SD_100_			(*P*-value)		
ML dose	ML_0_	ML_25_	ML_50_	ML_0_	ML_25_	ML_50_			
Treatments	SD_50_ML_0_	SD_50_ML_25_	SD_50_ML_50_	SD_100_ML_0_	SD_100_ML_25_	SD_100_ML_50_	SD	ML	SD*ML
Amylase (U/L)	15.50 ± 0.87	18.00 ± 0.58	20.50 ± 0.87	15.50 ± 1.44	17.00 ± 1.73	17.50 ± 3.18	0.351	0.156	0.671
Lipase (U/L)	22.50 ± 0.87^**c**^	31.00 ± 1.15^**ab**^	32.00 ± 1.73^**ab**^	17.50 ± 2.02^**d**^	28.00 ± 1.15^**b**^	33.00 ± 1.15^**a**^	0.065	0.000	0.136
Protease (U/L)	60.50 ± 0.64^**c**^	66.15 ± 1.07^**b**^	74.35 ± 2.68^**a**^	53.00 ± 1.15^**d**^	63.50 ± 1.44^**bc**^	68.00 ± 2.31^**b**^	0.002	0.000	0.364

Mean values in the same row with different superscript letters are significantly different (P < 0.05). Data represent mean ± SEM, n = 3 replicates

SD = stocking density, ML = melatonin.SD50ML0 = fish at stocking density of 50 fish 500L-1 fed BD without ML, SD50ML25 = fish at stocking density of 50 fish 500L-1 fed BD containing 25 mg kg-1 ML. SD50ML50 = fish at stocking density of 50 fish 500L-1 fed BD containing 50 mg kg-1 ML. SD100ML0 = fish at stocking density of 100 fish 500L-1 fed BD without ML. SD100ML50 = fish at stocking density of 100 fish 500L-1 fed BD containing 25 mg kg-1 ML. SD100ML50 = fish at stocking density of 100 fish 500L-1 fed BD containing 50 mg kg-1 ML

#### Growth hormone (GH) and insulin-like growth factor 1 (IGFI)

**Fig. 1:**
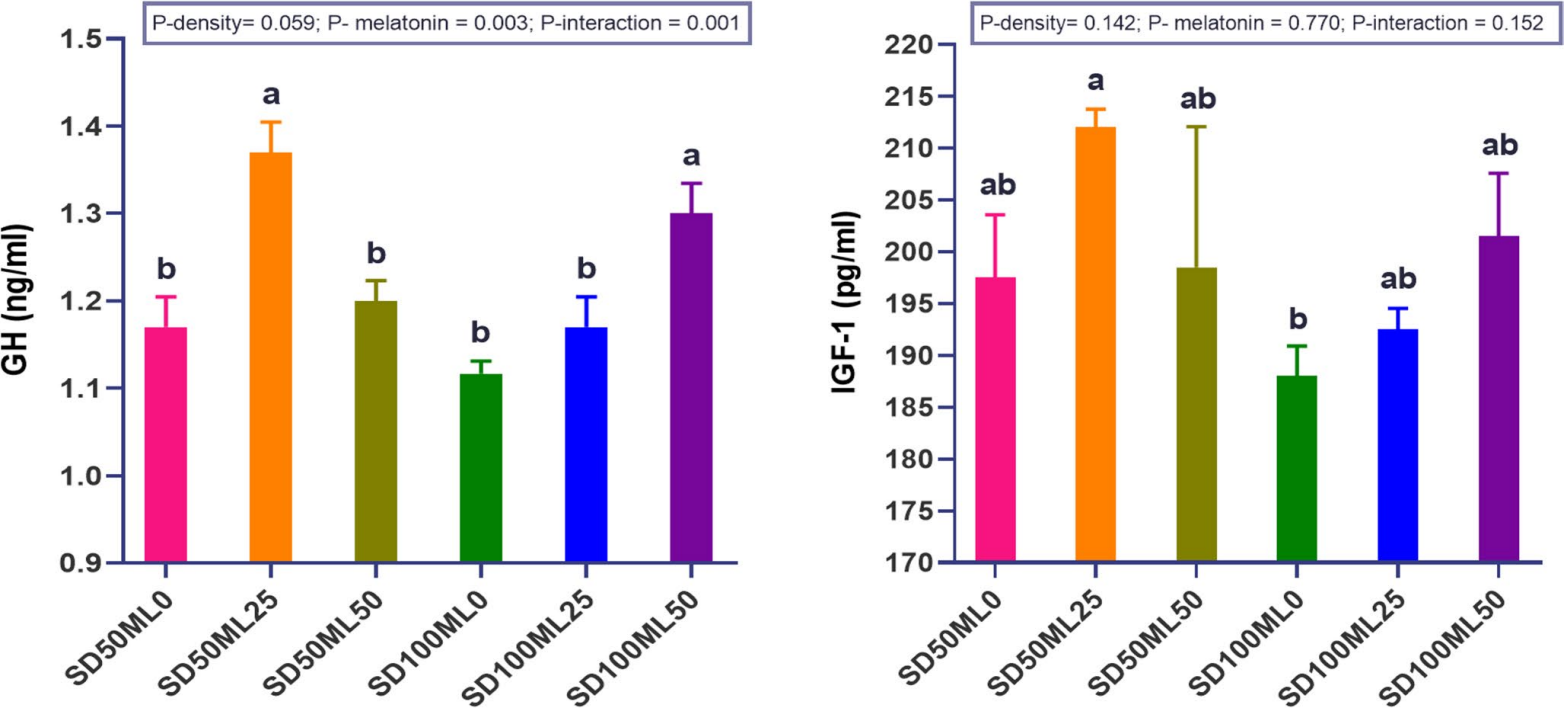
Serum growth hormone (GH) and Insulin-like growth factor 1 (IGF-I) of gilthead sea bream *Sparus aurata* fed different levels of melatonin under low and high stocking densities in groundwater conditions, for 90 days. where: SD_50_ML_0 _= fish at stocking density of 50 fish 500L^-1^fed BD without ML; SD_50_ML_25 _= fish at stocking density of 50 fish 500L^-1^fed BD containing 25 mg kg^-1^ ML; SD_50_ML_50 _= fish at stocking density of 50 fish 500L^-1^fed BD containing 50 mg kg^-1^ ML;SD_100_ML_0 _= fish at stocking density of 100 fish 500L^-1^fed BD without ML; SD_100_ML_50 _= fish at stocking density of 100 fish 500L^-1^fed BD containing 25 mg kg^-1^ ML; SD_100_ML_50 _= fish at stocking density of 100 fish 500L^-1^fed BD containing 50 mg kg^-1^ ML. SD = stocking density, ML= melatonin, BD = basal diet. 1 picograms (pg)/ml = 0.001 nanograms (ng)/ml. Data represent mean ± SEM, n = 3 replicates

Figure 1 shows how dietary ML and SD levels affect serum GH and IGF1 both on their own and in combination. The GH levels in the fish groups were significantly (P *< 0.05*) changed by the interaction effect between the ML dosage and SD. Both the fish fed 25 mg of ML under SD_50_ conditions and those fed 50 mg of ML under SD_100_ conditions presented higher GH levels than the other groups tested. Fish given ML doses of 50 mg and 25 mg under the SD_50_ and SD_100_, respectively, did not show improvements in GH levels, revealing the profound interaction between the ML dose and the SD level. On the other hand, the IGF1 values ​​were similar between the groups without significant differences except between the SD_50_ML_25_ (highest numerical value) and the SD_100_ML_0_ (lowest numerical value) groups. No significant difference (P *< 0.05*) in IGF1 levels was detected between the SD_50_ML_0_ and SD_100_ML_0_ treatments due to the independent effect of SD.

#### Serum liver enzymes, lipid profile and kidney health indicators

Table 6 shows the influences of ML and SD conditions on serum liver enzymes (ALT, AST, and ALP), kidney function indicators (ammonia, urea, and uric acid), and lipid profile (CHO, TG, LDL, and HDL) in *S. aurata* juveniles. Both factors greatly impacted most of the parameters (P *< 0.05*), often interactively (ML × SD). At both densities, ML administration led to reduced ALP levels in the fish compared to those fed the control diet. The interaction effect between the ML dose (25 mg) and SD resulted in significant reductions in AST, urea, and uric acid in the SD_50_ML_25_ group and urea in the SD_100_ML_25_ group, whereas the interaction effect between the ML (50 mg) and SD levels resulted in significant decreases in AST and urea (SD_50_ML_50_ group) and in AST, ALT, ammonia, urea, and uric acid in the SD_100_ML_50_ group compared with those in the control group. Compared with those in the other groups tested, serum lipids in *S. aurata* showed a dose-dependent interactive response between ML and SD, with higher levels of TG and HDL in the SD_100_ML_50_ group, CHO levels in the SD_50_ML_50_ and SD_100_ML_50_ groups, and LDL levels in the SD_100_ML_25_ group. ML administration at a dose of 50 mg under SD_100_ conditions resulted in significant decreases in the fish’s AST, ALP, ALT, ammonia, urea, and uric acid and significant increases in CHO, TG, and HDL while maintaining the same LDL value as in those fed a control diet. Increasing the ML dose to 50 mg under SD_50_ conditions did not improve liver or kidney function, as shown by the values ​​shown in Table 6. The independent effect of SD on liver enzymes and kidney function indices was significant, as evidenced by the higher values recorded in the SD_100_ML_0_ group than in the SD_50_ML_0_ group.

**Table 6:** Serum liver enzymes and kidney health indicators of gilthead sea bream Sparus aurata fed different levels of melatonin under different stocking densities in groundwater conditions for 90 days

SD	SD_50_			SD_100_			(*P*-value)		
ML dose	ML_0_	ML_25_	ML_50_	ML_0_	ML_25_	ML_50_			
Treatments	SD_50_ML_0_	SD_50_ML_25_	SD_50_ML_50_	SD_100_ML_0_	SD_100_ML_25_	SD_100_ML_50_	SD	ML	SD*ML
**Liver enzymes**									
AST (U/L)	170.5 ± 0.87^**a**^	91.5 ± 1.44^**c**^	95.0 ± 2.31^**c**^	153.5 ± 4.91^**b**^	145.5 ± 3.75^**b**^	77.5 ± 0.87^**d**^	0.108	0.002	0.183
ALT (U/L)	20.0 ± 1.15^**b**^	16.0 ± 1.73^**b**^	18.0 ± 1.73^**b**^	29.0 ± 0.58^**a**^	28.5 ± 0.87^**a**^	15.5 ± 1.44^**b**^	0.006	0.000	0.015
ALP (U/L)	296.0 ± 6.35^**a**^	182.5 ± 3.75^**d**^	191.0 ± 2.31^**d**^	279.5 ± 2.02^**b**^	241.5 ± 2.02^**c**^	165.5 ± 4.33^**e**^	0.000	0.002	0.001
**Kidney parameters**									
Ammonia (mg/dL)	61.0 ± 1.15^**ab**^	57.0 ± 1.15^**b**^	59.5 ± 1.44^**ab**^	64.5 ± 2.02^**a**^	60.5 ± 0.87^**ab**^	49.0 ± 2.31^**c**^	0.459	0.002	0.004
Urea nitrogen (mg/dL)	17.0 ± 0.58^**b**^	11.5 ± 0.87^**c**^	15.5 ± 1.15^**b**^	20.5 ± 0.87^**a**^	15.0 ± 1.15^**b**^	12.0 ± 0.58^**c**^	0.115	0.000	0.002
Uric acid (mg/dL)	1.46 ± 0.05^**b**^	1.18 ± 0.04^**c**^	1.10 ± 0.05^**c**^	1.93 ± 0.02^**a**^	1.89 ± 0.02^**a**^	1.17 ± 0.02^**c**^	0.000	0.000	0.000
**Lipid profile**									
Cholesterol (mg/dl)	216.5 ± 4.33^**d**^	219.5 ± 4.91^**d**^	298.0 ± 6.35^**a**^	251.5 ± 2.02^**c**^	269.0 ± 2.31^**b**^	303.5 ± 4.91^**a**^	0.000	0.000	0.001
Triglyceride (TG) (mg/dl)	378.5 ± 6.06^**b**^	372.0 ± 3.46^**b**^	389.5 ± 0.87^**b**^	374.5 ± 8.95^**b**^	382.0 ± 4.62^**b**^	406.5 ± 4.91^**a**^	0.108	0.002	0.183
HDL (mg/dl)	56.5 ± 0.87^**c**^	61.0 ± 1.15^**bc**^	60.5 ± 0.87^**bc**^	56.0 ± 1.15^**c**^	64.0 ± 1.73^**b**^	71.0 ± 2.89^**a**^	0.006	0.000	0.015
LDL (mg/dl)	71.0 ± 1.15^**c**^	72.0 ± 1.73^**c**^	83.0 ± 2.31^**b**^	85.5 ± 1.44^**b**^	93.5 ± 0.87^**a**^	87.5 ± 1.44^**b**^	0.000	0.002	0.001

Mean values in the same row with different superscript letters are significantly different (P < 0.05). Data represent mean ± SEM, n = 3 replicates

SD50ML0 = fish at stocking density of 50 fish 500L-1 fed BD without ML, SD50ML25 = fish at stocking density of 50 fish 500L-1 fed BD containing 25 mg kg-1 ML. SD50ML50 = fish at stocking density of 50 fish 500L-1 fed BD containing 50 mg kg-1 ML. SD100ML0 = fish at stocking density of 100 fish 500L-1 fed BD without ML. SD100ML50 = fish at stocking density of 100 fish 500L-1 fed BD containing 25 mg kg-1 ML. SD100ML50 = fish at stocking density of 100 fish 500L-1 fed BD containing 50 mg kg-1 ML. ALP (U/L) = alkaline phosphatase. ALT (U/L) = glutamic-pyruvic transaminase. AST (U/L) = aspartate amino transferase HDL (mg/dl) = high density lipoprotein LDL (mg/dl) = low density lipoprotein

#### Fish innate immunity parameters

The levels of innate immune markers in *S. aurata* fed different ML-supplemented diets under both SD conditions are presented in Table 7. The ML dose, SD level, and their interaction noticeably (*P < 0.05*) influenced the activity of most immune parameters. Under SD_50_ conditions, fish fed 25 mg of ML presented increased TP, ALB, GLO, IgM, and C4 levels compared with those fed a control diet or 50 mg of ML, whereas fish supplemented with 50 mg exhibited higher TP, ALB, and C4 compared with those in the control group (SD_50_ML_0_). Furthermore, under SD_100_ conditions, fish fed 25 and 50 mg ML maintained higher (P *< 0.05*) levels of TP, GLO, and C3 than those fed a control diet, whereas the IgM and C4 values ​​were significantly greater in the fish fed 50 mg ML than in those fed 25 mg ML or a control diet. ML addition at various doses did not improve the C3 and ALB values ​​under SD_50_ and SD_100_ conditions, respectively. SD alone had a significant impact and altered the values ​​of TP, ALB, and C4 between the SD_50_ML_0_ and SD_100_ML_0_ groups.

**Table 7:** Innate immune parameters of gilthead sea bream Sparus aurata fed different levels of melatonin under different stocking densities in groundwater conditions for 90 days

SD	SD_50_			SD_100_			(*P*-value)		
ML dose	ML_0_	ML_25_	ML_50_	ML_0_	ML_25_	ML_50_			
Treatments	SD_50_ML_0_	SD_50_ML_25_	SD_50_ML_50_	SD_100_ML_0_	SD_100_ML_25_	SD_100_ML_50_	SD	ML	SD*ML
Total protein, (mg/dL)	6.465 ± 0.10^**c**^	7.430 ± 0.16^**a**^	7.065 ± 0.08^**b**^	7.010 ± 0.01^**b**^	7.565 ± 0.08^**a**^	7.360 ± 0.02^**a**^	0.001	0.000	0.102
Albumin, (mg/dL)	2.210 ± 0.05^**c**^	2.655 ± 0.03^**a**^	2.525 ± 0.04^**b**^	2.480 ± 0.01^**b**^	2.555 ± 0.03^**ab**^	2.500 ± 0.01^**b**^	0.098	0.000	0.000
Globulin, (mg/dL)	4.255 ± 0.15^**c**^	4.775 ± 0.13^**ab**^	4.540 ± 0.03^**bc**^	4.530± 0.02^**bc**^	5.010 ± 0.11^**a**^	4.860 ± 0.03^**a**^	0.004	0.001	0.904
Compliment C3 (mg/dL)	35.50 ± 2.02^**bc**^	41.50 ± 1.44^**ab**^	35.00 ± 2.31b^**c**^	31.00 ± 1.73^**c**^	42.00 ± 2.89^**ab**^	46.50 ± 2.60^**a**^	0.193	0.005	0.011
Compliment C4 (mg/dL)	3.620 ± 0.10^**e**^	4.835 ± 0.16^**c**^	4.125 ± 0.05^**d**^	5.045 ± 0.04^**bc**^	5.180 ± 0.06^**b**^	6.040 ± 0.15^**a**^	0.00	0.000	0.000
Immunoglobin (mg/ml)	10.95 ± 0.20^**c**^	13.60 ± 0.40^**a**^	11.15 ± 0.20^**bc**^	10.80 ± 0.58^**c**^	11.25 ± 0.66^**bc**^	12.50 ± 0.40^**ab**^	0.311	0.014	0.004

Mean values in the same row with different superscript letters are significantly different (P < 0.05). Data represent mean ± SEM, n = 3 replicates

SD = stocking density, ML = melatonin. SD50ML0 = fish at stocking density of 50 fish 500L-1 fed BD without ML, SD50ML25 = fish at stocking density of 50 fish 500L-1 fed BD containing 25 mg kg-1 ML. SD50ML50 = fish at stocking density of 50 fish 500L-1 fed BD containing 50 mg kg-1 ML. SD100ML0 = fish at stocking density of 100 fish 500L-1 fed BD without ML. SD100ML50 = fish at stocking density of 100 fish 500L-1 fed BD containing 25 mg kg-1 ML. SD100ML50 = fish at stocking density of 100 fish 500L-1 fed BD containing 50 mg kg-1 ML

#### Antioxidant status

**Fig. 2:**
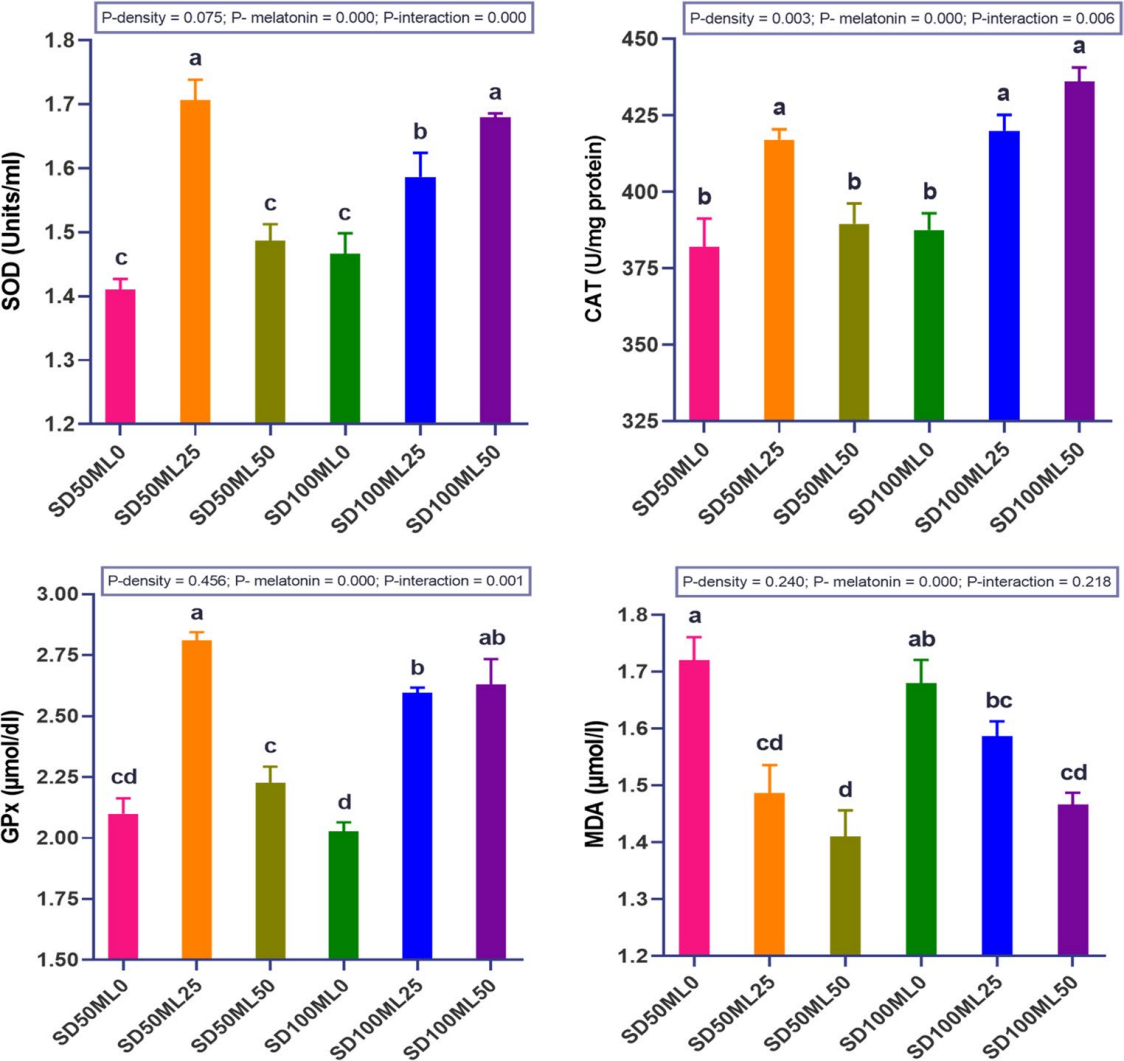
Antioxidant parameters of gilthead sea bream *Sparus aurata* fed with different levels of melatonin under low and high stocking densities in groundwater conditions, for 90 days. where: SD_50_ML_0 _= fish at stocking density of 50 fish 500L^-1^fed BD without ML; SD_50_ML_25 _= fish at stocking density of 50 fish 500L^-1^fed BD containing 25 mg kg^-1^ ML; SD_50_ML_50 _= fish at stocking density of 50 fish 500L^-1^fed BD containing 50 mg kg^-1^ ML; SD_100_ML_0 _= fish at stocking density of 100 fish 500L^-1^fed BD without ML; SD_100_ML_50 _= fish at stocking density of 100 fish 500L^-1^fed BD containing 25 mg kg^-1^ ML; SD_100_ML_50 _= fish at stocking density of 100 fish 500L^-1^fed BD containing 50 mg kg^-1^ ML. SD = stocking density, ML = melatonin, BD = basal diet, SOD = Superoxide dismutase activity, GPx = glutathione peroxidase, CAT = Catalase, MDA = malondialdehyde. Data were analyzed with two-way ANOVA and one-way ANOVA, Tukey test, n = 3. Data represent mean ± SEM, n = 3 replicates

Figure 2 presents the liver antioxidative enzymes in *S. aurata* fed a diet supplemented with various ML doses under different SD conditions. ML dose, SD level, and their interaction significantly (P *< 0.05*) altered antioxidant activity. In comparison to the control diet, the incorporation of ML at dosages of 25 and 50 mg under SD_100_ conditions, as well as 25 mg under SD_100_ conditions, significantly (P *< 0.05*) enhanced the antioxidant capacity (SOD, CAT, and GPx) of the fish. Increasing the ML dosage to 50 mg in the diet of fish raised under SD_50_ conditions did not improve the liver’s SOD, CAT, or GPx status when compared to the control diet. In contrast, the control groups (SD_50_ML_0_ and SD_100_ML_0_) had a significantly higher level of MDA than the fish groups that were fed diets that were higher in ML. SD alone exerted no significant effect on the levels of antioxidant enzymes.

#### Serum immune Proinflammatory cytokine Interleukin 1β (IL- 1β)

**Fig. 3:**
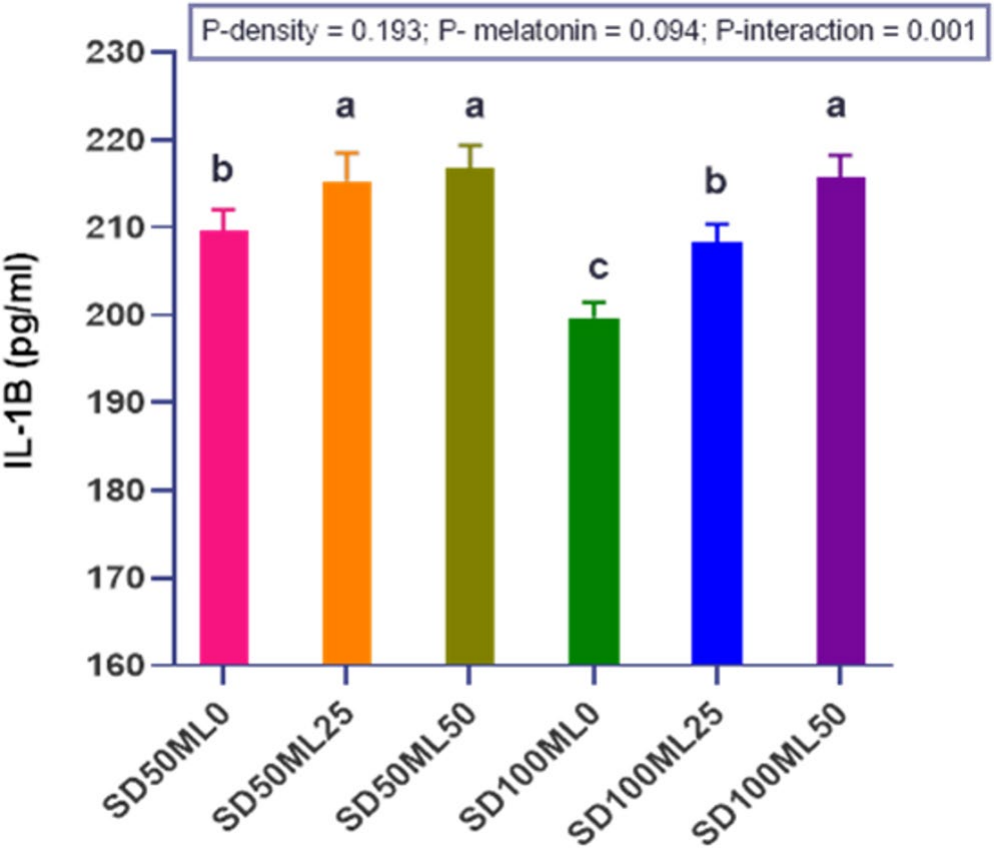
Serum immune pro-inflammatory cytokine interleukin-1β (IL-1β) in gilthead sea bream *Sparus aurata* fed different levels of melatonin under low and high stocking densities under groundwater conditions, for 90 days. where: SD_50_ML_0 _= fish at stocking density of 50 fish 500L^-1^fed BD without ML; SD_50_ML_25 _= fish at stocking density of 50 fish 500L^-1^fed BD containing 25 mg kg^-1^ ML; SD_50_ML_50 _= fish at stocking density of 50 fish 500L^-1^fed BD containing 50 mg kg^-1^ ML; SD_100_ML_0 _= fish at stocking density of 100 fish 500L^-1^fed BD without ML; SD_100_ML_50 _= fish at stocking density of 100 fish 500L^-1^fed BD containing 25 mg kg^-1^ ML; SD_100_ML_50 _= fish at stocking density of 100 fish 500L^-1^fed BD containing 50 mg kg^-1^ ML. SD = stocking density, ML = melatonin, BD = basal diet. pg/ml = 0.001 ng/ml. 1 picograms (pg)/ml = 0.001 nanograms (ng)/ml. Data represent mean ± SEM, n = 3 replicates.

Figure 3 illustrates the levels of serum immune proinflammatory cytokines (IL-1β) in *S. aurata* fed different levels of ML under both SD conditions in groundwater. Under both densities, ML administration displayed a considerable (P *< 0.05*) increase in IL-1β levels in fish-fed diets supplemented with varying doses of ML compared to those fed a control diet. Under SD_50_ conditions, increasing the ML dose to 50 mg (SD_50_ML_50_ group) in the diet did not lead to a further increase in the IL-1β levels in the fish, unlike under SD_100_ conditions, where a significant increase in the fish IL-1β levels (SD_100_ML_50_ group) was recorded compared with those fed the 25 mg ML (SD_100_ML_250_ group). SD alone exerted a substantial effect, as IL-1β levels were significantly (P *< 0.05*) reduced in the SD_100_ML_0_ group compared to the SD_50_ML_0_ group.

#### Stress and other biochemical markers in *Sparus aurata*

**Fig. 4:**
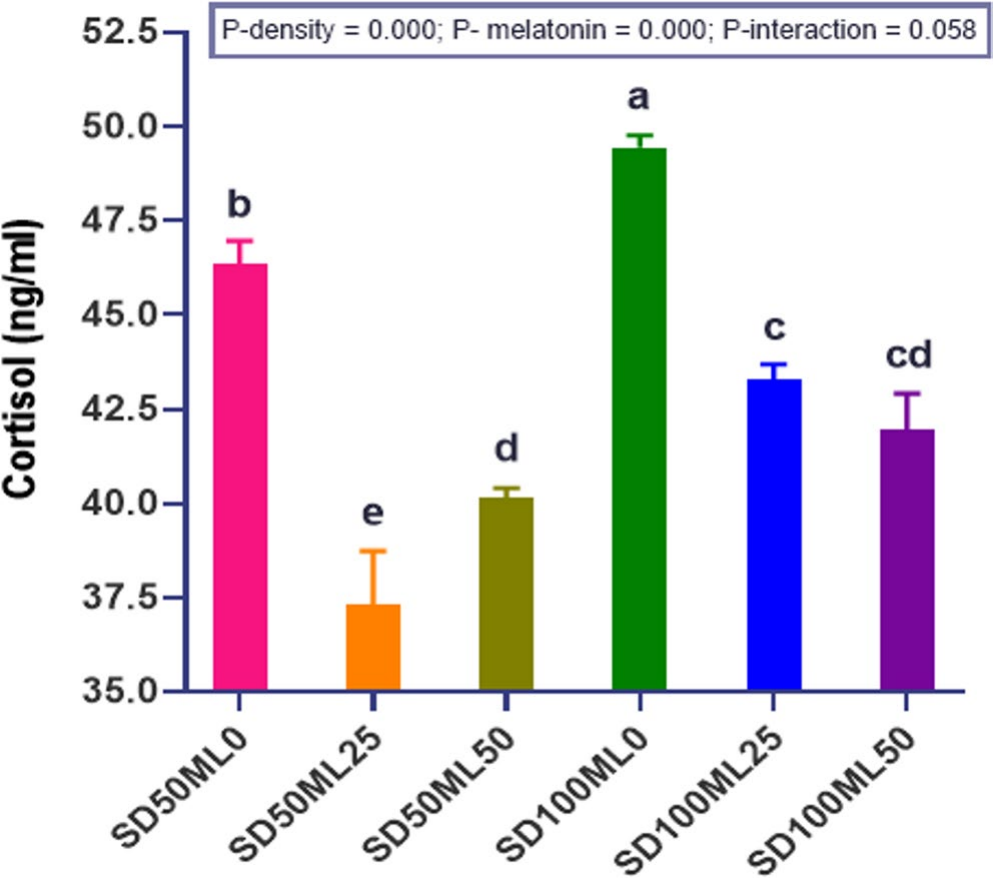
Cortisol stress biomarkersof gilthead sea bream *Sparus aurata* fed different levels of melatonin under low and high stocking densities in groundwater conditions, for 90 days. where: SD_50_ML_0 _= fish at stocking density of 50 fish 500L^-1^fed BD without ML; SD_50_ML_25 _= fish at stocking density of 50 fish 500L^-1^fed BD containing 25 mg kg^-1^ ML; SD_50_ML_50 _= fish at stocking density of 50 fish 500L^-1^fed BD containing 50 mg kg^-1^ ML; SD_100_ML_0 _= fish at stocking density of 100 fish 500L^-1^fed BD without ML; SD_100_ML_50 _= fish at stocking density of 100 fish 500L^-1^fed BD containing 25 mg kg^-1^ ML; SD_100_ML_50 _= fish at stocking density of 100 fish 500L^-1^fed BD containing 50 mg kg^-1^ ML. SD = stocking density, ML = melatonin, BD =basal diet. Data were analyzed with two-way ANOVA and one-way ANOVA, Tukey test, n = 3. Data represent mean ± SEM, n = 3 replicates

**Fig. 5:**
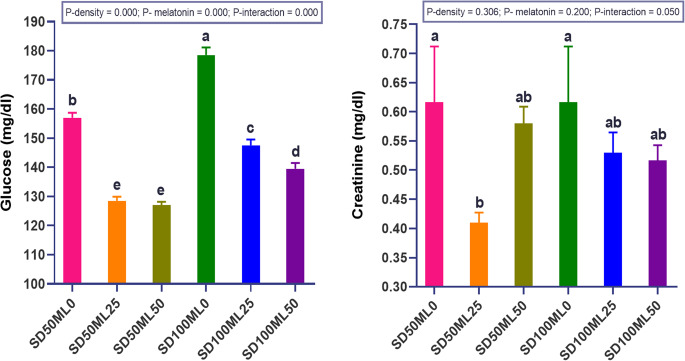
Glucose and creatinine biochemical markersof gilthead sea bream *Sparus aurata* fed different levels of melatonin under low and high stocking densities in groundwater conditions, for 90 days. where: SD_50_ML_0 _= fish at stocking density of 50 fish 500L^-1^fed BD without ML; SD_50_ML_25 _= fish at stocking density of 50 fish 500L^-1^fed BD containing 25 mg kg^-1^ ML; SD_50_ML_50 _= fish at stocking density of 50 fish 500L^-1^fed BD containing 50 mg kg^-1^ ML; SD_100_ML_0 _= fish at stocking density of 100 fish 500L^-1^fed BD without ML; SD_100_ML_50 _= fish at stocking density of 100 fish 500L^-1^fed BD containing 25 mg kg^-1^ ML; SD_100_ML_50 _= fish at stocking density of 100 fish 500L^-1^fed BD containing 50 mg kg^-1^ ML. SD = stocking density, ML = melatonin, BD =basal diet. Data were analyzed with two-way ANOVA and one-way ANOVA, Tukey test, n = 3. Data represent mean ± SEM, n = 3 replicates

Adding ML to the fish’s diet significantly (P *< 0.05*) lowered serum stress marker levels (cortisol, COR) in the serum compared to the control diet. This happened in both SD_50_ and SD_100_ conditions (Fig. 4). The reduction in cortisol concentration at low density (SD_50_) was more pronounced at low melatonin concentration (25 mg kg⁻¹ ML), but the effect was more significant at high-density (SD_100_) with high melatonin concentration (50 mg kg⁻¹ ML). Figure 5 illustrates the levels of serum creatinine and glucose. Fish in all ML-supplemented groups had lower amounts of CRE compared to fish fed a control diet. The SD_50_ML_25_ group had the lowest amount of CRE, which was significantly different from the control groups (SD_50_ML_0_ and SD_100_ML_0_). Increasing the ML dose to 50 mg in the fish diet did not result in further decreases in the serum GLU, and CRE levels when the fish were grown under SD_50_ conditions, as well as COR and CRE levels under SD_100_ conditions. SD alone had a meaningful impact (P *< 0.05*), as COR and GLU levels increased significantly in the SD_100_ML_0_ group compared with those in the SD_50_ML_0_ group, while both groups maintained the same CRE level.

### Histopathological alterations in the fish liver and intestine

**Fig. 6:**
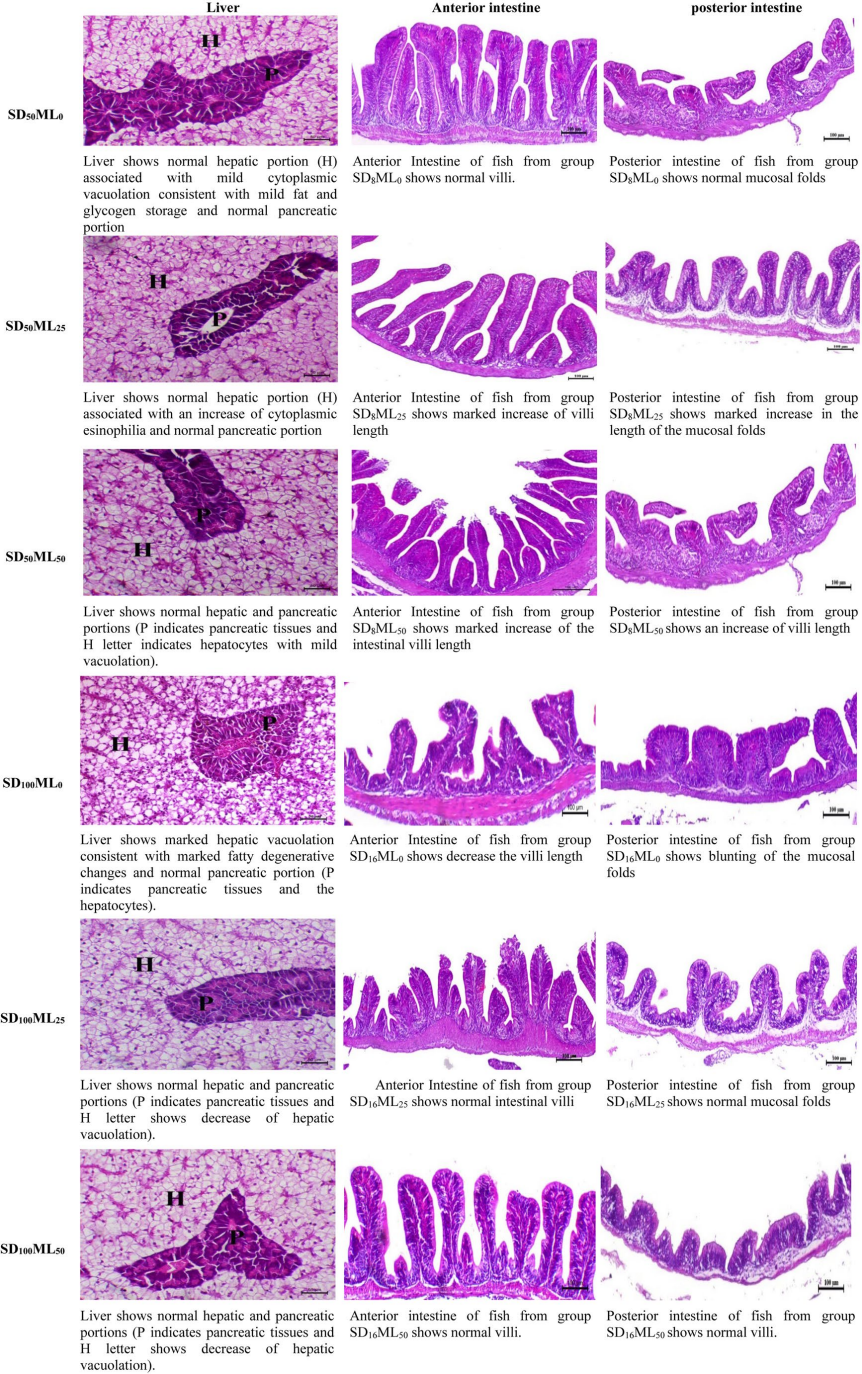
Histological changes in the liver and intestine (anterior and posterior portions) of gilthead sea bream *Sparus aurata* fed different levels of melatonin under low and high stocking densities in groundwater conditions, for 90 days. Hepatic vacuolation (H letter), normal pancreatic portions (P letter); Liver stained with H&E, X50, bar = 50 µm. Intestine stained with H&E, X100, bar = 100 µm. Where: SD_50_ML_0_ = fish at stocking density of 50 fish 500L^-1^fed BD without ML; SD_50_ML_25 _= fish at stocking density of 50 fish 500L^-1^fed BD containing 25 mg kg^-1^ ML; SD_50_ML_50_ = fish at stocking density of 50 fish 500L^-1^fed BD containing 50 mg kg^-1^ ML; SD_100_ML_0_ = fish at stocking density of 100 fish 500L^-1^fed BD without ML; SD_100_ML_50_ = fish at stocking density of 100 fish 500L^-1^fed BD containing 25 mg kg^-1^ ML; SD_100_ML_50_ = fish at stocking density of 100 fish 500L^-1^fed BD containing 50 mg kg^-1^ ML. SD = stocking density, ML = melatonin, BD = basal diet

Histomorphological modifications in the liver and intestine (anterior and posterior parts) of *S. aurata* fed three levels of ML at various SD conditions are shown in Fig. 6. Microscopic photographs of the livers of the fish in the six groups revealed normal hepatic and pancreatic parts associated with mild cytoplasmic vacuolation and mild fat (SD_50_ML_0_ group), increased cytoplasmic eosinophilia (SD_50_ML_25_ group), hepatocytes with mild vacuolation (SD_50_ML_50_ group), marked hepatic vacuolation consistent with marked fatty degenerative changes (SD_100_ML_0_ group), and a decrease in hepatic vacuolation in the SD_100_ML_25_ and SD_100_ML_50_ groups. ML supplementation improved fish livers, as evidenced by decreased hepatic vacuolization and fatty degenerative alterations in the ML-supplemented fish compared with those fed the control diet (SD_50_ML_0_ and SD_100_ML_0_ groups). Compared with those fed a 50 mg dose, the fish fed a low dose of ML (25 mg) presented healthier liver tissues under SD_50_ conditions, whereas under SD_100_ conditions, both ML doses (25 and 50 mg) maintained healthy livers with reduced vacuolization. Fish fed a control diet under SD_100_ conditions (SD_100_ML_0_ group) showed significantly increased vacuolization and fatty degeneration in liver tissues compared to those fed the same diet under SD_50_ conditions (SD_50_ML_0_ group), demonstrating the significant impact of SD on the liver health in *S. aurata*.

According to microscopic examination of the foregut and hindgut of *S. aurata* (Fig. 6; Table 8), the integration of ML and SD conditions significantly influences the histological measures of the fish intestine. In comparison to fish fed a control diet, fish given ML supplementation under both SD_50_ and SD_100_ conditions showed a significant increase in the number of goblet cells and villus length across the anterior and posterior intestines. Under SD_50_ conditions, the fish fed both ML doses (25 and 50 mg) presented the greatest increase (P *< 0.05*) in the number of goblet cells, whereas the fish fed ML 25 mg displayed the highest villi length across both the foregut and hindgut compared to the other groups tested. Furthermore, increasing the ML dose to 50 mg under SD_100_ conditions resulted in significant improvements in the goblet cell number (foregut) and villus length (hindgut). SD level considerably impacted the intestinal dimensions (anterior and posterior intestines), as indicated by decreased goblet cell numbers, villus length, and increased intervillous space in the SD_100_ML_0_ group compared to the SD_50_ML_0_ group.

**Table 8:** Histomorphometric changes in the intestine (anterior and posterior) of gilthead sea bream Sparus aurata fed different levels of melatonin under different stocking densities in groundwater conditions for 90 days

SD	SD_50_				SD_100_			(*P*-value)		
ML dose	Parameter	ML_0_	ML_25_	ML_50_	ML_0_	ML_25_	ML_50_			
Treatments		SD_50_ML_0_	SD_50_ML_25_	SD_50_ML_50_	SD_100_ML_0_	SD_100_ML_25_	SD_100_ML_50_	SD	ML	SD*ML
Anterior	Villi length, µm	217.92±5.50^**c**^	345.46±11.69^**a**^	275.18±13.71^**b**^	150.41±21.90^**d**^	197.80±6.55^**c**^	237.44±9.78^**bc**^	0.000	0.000	0.003
	Villi width, µm	63.38±4.89	82.47±14.07	70.92±14.14	73.26±6.09	81.77±6.59	89.40±3.95	0.247	0.310	0.599
	Inter villi space, µm	95.95±8.09^**bc**^	53.59±7.73^**e**^	72.84±4.76^**de**^	138.62±9.07^**a**^	107.30±5.09^**b**^	84.19±6.94^**cd**^	0.000	0.000	0.030
	Goblet cells, #/mm^2^	33.67±1.20^**b**^	45.67±2.60^**a**^	43.67±2.19^**a**^	18.33±0.88^**d**^	26.00±2.08^**c**^	32.00±2.08^**b**^	0.000	0.000	0.160
Posterior	Villi length, µm	109.77±6.38^**d**^	253.45±7.61^**a**^	209.39±9.01^**b**^	73.87±5.79^**e**^	125.89±7.44^**d**^	177.57±6.38^**c**^	0.000	0.000	0.000
	Villi width, µm	72.64±8.21	83.96±9.84	71.13±12.31	74.22±7.02	95.03±9.04	107.57±28.23	0.189	0.416	0.295
	Inter villi space, µm	120.70±9.81^**b**^	54.59±9.32^**c**^	80.96±9.42^**bc**^	180.24±11.56^**a**^	94.72±22.08^**bc**^	89.94±5.63^**bc**^	0.004	0.000	0.164
	Goblet cells, #/mm^2^	22.33±1.45^**c**^	31.33±1.86^**a**^	29.33±2.33^**ab**^	11.33±1.20^**d**^	22.33±1.45^**c**^	25.67±1.45^**bc**^	0.000	0.000	0.116

Mean values in the same row with different superscript letters are significantly different (P < 0.05). Data represent mean ± SEM, n = 3 replicates

SD = stocking density, ML = melatonin. SD50ML0 = fish at stocking density of 50 fish 500L-1 fed BD without ML, SD50ML25 = fish at stocking density of 50 fish 500L-1 fed BD containing 25 mg kg-1 ML. SD50ML50 = fish at stocking density of 50 fish 500L-1 fed BD containing 50 mg kg-1 ML. SD100ML0 = fish at stocking density of 100 fish 500L-1 fed BD without ML. SD100ML50 = fish at stocking density of 100 fish 500L-1 fed BD containing 25 mg kg-1 ML. SD100ML50 = fish at stocking density of 100 fish 500L-1 fed BD containing 50 mg kg-1 ML

## Discussion

Intensive aquaculture practices may negatively impact environmental conditions and promote production-related diseases. This research explored the growth and physiological responses of *S. aurata* cultured intensively in groundwater, with an emphasis on the effects of fish density as a stressor and the application of ML as a feed additive to reduce stress at high densities and improve culture conditions.

### Water quality in rearing tanks

In intensive aquaculture systems, the accumulation of inorganic nitrogen is the main issue influencing water quality (Xu et al. 2021; Abdel-Rahim et al. 2025), with established harmful effects of NH_₃_ (> 0.1 mg L⁻¹) (Qiao et al. 2006) and NO_₂_ (Ciji and Akhtar 2020) in fish farming media, in addition to the increased oxygen demand necessitated by increased stocking densities (Li et al. 2021). The findings of this study clearly reveal the significant role of ML in reducing nitrogen byproducts (TAN, NH_3_, and NO_2_) and numerically increasing oxygen levels in the tanks of the ML-augmented groups, compared with those in the control group. This outcome can be interpreted as a direct effect of improved feed efficiency and protein metabolism, diminished organic matter load, and a parallel reduction in fish movement. The current study’s findings are in line with the sole investigation into how ML can enhance water quality (Adah et al. 2023). Adah et al. (2023) reported that giving ML to catfish for one month greatly improved their water quality, with oxygen level increasing to 54.8% (4.66 ppm vs. 3.01 ppm), ammonia level decreasing to 82.3% (0.03 ppm vs. 0.17 ppm), and nitrite level declining to 55.6% (0.04 ppm vs. 0.09 ppm) after 100-kilometer transportation compared to without ML. Adding ML to fish feed reduces fish movement, swimming speed, and oxygen consumption (Sánchez-Vázquez et al. 2019). As the SD increases, fish movement, respiration, and waste excretion increase (Gatica et al. 2008), especially in the groups not fed the ML. This expounds the higher nitrogenous waste and lower dissolved oxygen levels recorded in the control groups (SD_50_ML_0_ and SD_100_ML_0_) in this study. Rubio-Gracia et al. (2020) revealed that oxygen consumption has a robust positive correlation with fish swimming speed and that elevated ammonia concentrations are associated with high densities. Biswas et al. (2006) reported that the correlation between ammonia content and fish density is mostly linear. Furthermore, Ott et al. (2024) found that nitrite and nitrate concentrations were higher in ponds with higher stocking densities, suggesting that surplus nitrogen wastes speed up nitrification. The administration of ML at suitable doses to fish feed has sedative effects (Sánchez-Vázquez et al. 2019). A primary favorable outcome is a substantial reduction in several harmful stressors in fish, leading to enhanced vital processes, including improved feed metabolism and a subsequent decrease in nitrogen waste generation.

### GH, IGF1 and Growth performances

Growth is a polygenic characteristic influenced by environmental factors, with the most significant genes being GH and IGF1, which form the foundation of the hypothalamic–pituitary–somatotropic (HPS) axis (Elhetawy et al. 2025). Thus, controlling the influence of environmental and dietary factors on growth-related GH and IGF1 genes presents a significant opportunity to improve fish performance and welfare (Triantaphyllopoulos et al. 2020). Here, our findings disclosed that the expression of GH and IGF1, growth parameters, and feeding efficacy of *S. aurata* were enhanced when fed diets augmented with ML compared to the control groups across both SD_50_ and SD_100_ conditions. This increase occurred in a dose-density-dependent manner, indicating a profound interaction between the ML dose and SD level under groundwater conditions. Researchers have previously reported that the failure of dietary ML to enhance the growth performance of some fish species is due to loss of appetite (Conde-Sieira et al. 2014; Amri et al. 2020; Veisi et al. 2021). Other studies have also shown that the ability of ML to hinder feeding in poultry is due to its soothing influence on locomotor behavior (Murakami et al. 2001; Zhdanova et al. 2002). This, however, contradicts the findings of the present investigation as well as numerous earlier reports on the beneficial effects of ML on aquatic animal growth (Yang et al. 2023; Lv et al. 2024; Ye et al. 2024). This discrepancy suggests that the integrated action of GH/IGF1 and thyroid hormones/IGF1 may control the daily routine of fish growth, and ML may play an important role in this approach, which varies from species to species (Rizky et al. 2024). Moreover, external factors, such as diet, temperature, photoperiod, salinity and SD, can influence GH and IGFI expression, causing growth rates to differ between species through changes in the biological cycles of these genes (Triantaphyllopoulos et al. 2020). Furthermore, the growth rate of a single fish species can vary depending on the culture technique applied, daily practices, and farming conditions (Lotfy et al. 2023).

The higher levels of GH and IGF1 in *S. aurata* that were fed ML-enriched diets are similar to what other studies have found (Falcón et al. 2003; Mhalhel et al. 2020; Rizky et al. 2024). The superior growth characteristics and feed utilization indices align with other findings (Li et al. 2023; Yang et al. 2023; Lv et al. 2024; Ye et al. 2024). The improved growth performance could be attributed to enhanced digestive enzyme activity and gastrointestinal health due to the positive effects of ML on the structure, diversity, and abundance of the gut flora, thus promoting nutrient absorption (Lv et al. 2024). The improvement in GH and IGF in ML fish could be attributed to the role of ML in controlling the synthesis of hormones/peptides (including growth- and stress-influencing hormones) within the circadian system (Rizky et al. 2024), suggesting that ML supplementation enhances GH/IGF1 and suppresses stress hormones (Triantaphyllopoulos et al. 2020). Stressors such as permanent light have been found to suppress the ML circadian rhythm (Falcon et al. 2010). In this regard, Chen et al. (2025) found that dietary ML at 3:00 PM had a stronger effect on the physiology of crayfish (*Procambarus clarkii*) than feeding at 3:00 AM, and markedly enhanced the expression of circadian clock-related genes, influencing mRNA expression levels at various times, with a greater upregulation rate at 3:00 PM. However, elevated melatonin supplementation resulted in dramatically reduced mRNA levels of these genes. The current findings suggest that ML-enriched feed may support synchronization of circadian gene expression and maintenance of circadian balance in *S. aurata* across higher SD levels and groundwater conditions.

### Body composition

Dietary supplementation with ML increased the dry matter and crude protein contents, decreased the crude lipid content, and kept the crude ash constant in *S. aurata* demonstrating that ML can enhance the carcass’s nutritional value in a dose-density-dependent manner under varying SD in groundwater conditions. The results for increased crude protein and dry matter contents as well as constant ash content are consistent with those of *Catla catla* fingerlings fed increasing levels of dietary tryptophan up to 2.3 g kg/diet (crude protein) and up to 3.4 g kg (dry matter) (Zehra and Khan 2015). Here, the lower crude fat content corresponds to that reported for *P. vannamei* fed ML supplements of 41.2, 82.7, 165.1, and 329.2 mg/kg diet (Ye et al. 2024). The fact that the ML-added groups’ fish carcasses had less fat suggests that ML may speed up the oxidative breakdown of lipids in *S. aurata*, which would change the energy balance to favor lipid metabolism over other energy sources. Given the high digestibility of lipids, this suggests that *S. aurata* preferentially solubilizes fats to obtain the necessary energy (Beseres et al. 2005). The higher crude protein in the fish’s muscles in the ML-augmented groups may be because their protein metabolism is better and they make more endogenous enzymes that help the pancreas release cholecystokinin and exocrine pancreatic secretion. This changes the way the digestive tract works and makes it easier for food and supplements to be broken down and absorbed (Elhetawy et al. 2024).

### Digestive enzymes

*S. aurata* fed ML supplements under both densities revealed significant improvements in digestive enzyme (lipase and protease) activity, indicating improved protein digestibility. Increasing the ML dose was associated with improved lipase activity under SD_100_ and protease activity under SD_50_ conditions, while no effect on amylase activity was recorded among the groups tested. These findings revealed that the effect of the optimum ML dose and SD on digestive enzymes was dose- and density-dependent. The enhanced activity of digestive enzymes can be ascribed to several functions performed by ML: enhancing digestion and nutritional absorption by extending “food transit time” (Velarde et al. 2009) and diminishing intestinal motility through AANAT-mediated acetylation of key motility factors, serotonin and dopamine (Nisembaum et al. 2013); (2) boosting hormones and peptides associated with gastrointestinal physiology, such as leptin, ghrelin, and peptides (Aydin et al. 2008; Taheri et al. 2019); (3) relaxing smooth muscle in the gastrointestinal tract by preventing contraction through gastric and ileal (Acharyya et al. 2021); and (4) enhancing the diversity, structure and balance of the gut microbiota (Yin et al. 2020; Zhang et al. 2021). In line with the present findings, oral administration of ML to rainbow trout exposed to high SD stress improved lipase, amylase, and protease activities in stressed fish compared with those in the control group (normal SD) (Conde-Sieira et al. 2014). Similarly, previous studies have shown the positive effects of exogenous ML on digestive enzyme activity in various aquatic animals, including the rice field eel *Monopterus albus* (Lv et al. 2024), Atlantic salmon *Salmo salar* (Mardones et al. 2022), and *Eriocheir sinensis* (Zhang et al. 2018). These authors attributed the enhanced digestive enzyme activity to the partial role of ML in influencing gut health by improving immunity and restructuring the intestinal microbiota, leading to improved nutrient absorption and maintenance of intestinal barrier integrity.

### Lipid profile, liver enzymes and kidney metabolism

AST, ALT, ALP, ammonia, urea, and uric acid are enzymes and physiological markers commonly found in various tissues, such as fish liver, gills, blood, muscle, and kidney (Rather 2015; Veisi et al. 2021). Straining these parameters uncovered clear evidence of the improved health status and physiological function of *S. aurata* with ML supplementation. Moreover, screening of serum lipids (CHO, TG, HDL, and LDL) in *S. aurata* revealed apparent evidence of enhanced metabolism with ML supplementation as well. ML administration (25 mg and 50 mg/kg diet) improved most of the liver and kidney indicators and serum lipids in fish under both densities. Increasing ML to 50 mg/kg under SD_100_ conditions enhanced liver and kidney functions, as evidenced by decreased values of their markers, along with decreased LDL and improved HDL. HDL has many physiological roles, such as promoting the efflux of excess cholesterol from peripheral tissues to the liver for disposal and thus protecting against cardiovascular diseases, modulating endothelial function, and inhibiting the oxidative modification of LDL (Harel et al. 1990; Norata et al. 2012; Andreeva et al. 2020). Consistent with these findings, previous studies have documented the role of dietary ML in improving liver acetylcholinesterase and ALP in *S. aurata* (Amri et al. 2020), liver AST in *Oreochromis niloticus* (Veisi et al. 2021), kidney health in the Prussian carp *Carassius gibelio* against cadmium exposure (Drąg-Kozak et al. 2021), and lipid metabolism in *P. vannamei* (Ye et al. 2024). Dietary ML could explain these improvements by increasing the production of ML in the pineal gland which regulates various functions, including circadian rhythms (Yang et al. 2020; Veisi et al. 2021), the immunological system, and the central nervous system (Yang et al. 2020; Ye et al. 2024). It facilitates the ability of aquatic animals to manage environmental stressors (Sainath et al. 2013; Ye et al. 2024). The increase in serum CHO and TG in the fish-fed ML, especially at the high dose (50 mg), could be attributed to the relaxation of fish with reduced influence of stressors, resulting in lower energy intake even under higher SD conditions (SD_100_ML_50_ group).

### Innate immunity and antioxidant capacity

Intensively farmed marine species are more susceptible to environmental stresses, which negatively impact the immune defense and produce excess amounts of reactive oxygen (RO) during metabolic processes, leading to oxidative stress (OS) (Sallam et al. 2025; Abdel-Rahim et al. 2025). OS occurs when the generation of RO exceeds the cellular antioxidant capacity, leading to structural damage induced by RO (Van Horssen et al. 2008). Dietary administration of ML is conveyed to modulate the immune response of *S. aurata* (Cuesta et al. 2008), *Salmo salar* and *Oncorhynchus kisutch* (Mardones et al. 2022), both the antioxidant capacity and innate immunity of *Eriocheir sinensis* (Zhang et al. 2019), and *Litopenaeus vannamei* (Li et al. 2023). In the present study, dietary ML significantly enhanced the levels of innate immune markers (TP, ALB, GLO, IgM, C3, and C4) of *S. aurata* grown under SD_50_ and SD_100_ conditions, revealing a profound effect between the ML dosage and the SD level. Our investigation also demonstrated that the activities of CAT, SOD, and GPx displayed considerable increases in fish fed 50 mg/mL, and the MDA level markedly decreased compared with that in the control group under SD_100_ conditions. On the other hand, fish fed 25 mg of ML under SD_50_ conditions showed significant improvements in their CAT, SOD, and GPx activities and notable decreases in their MDA levels, whereas increasing the ML dose to 50 mg did not lead to any improvement in their antioxidant status. In line with these findings, crayfish *Cherax destructor* fed increasing ML concentrations (0, 20, 40, 80, 160, and 320 mg/kg diet) tended to first improve and then deteriorate in terms of antioxidant activity, with significant increases in total antioxidant capacity and glutathione reductase (80 mg/kg), maximum SOD (160 mg/kg), and reduced glutathione (20, 40, and 80 mg/kg) contents and lower MDA (160 mg/kg) contents than those fed the control diet (Yang et al. 2023). Similarly, dietary ML addition significantly improved SOD activity in common carp (Ghodrati Azadi et al. 2013) and SOD and CAT activity in *O. mykiss* (Gülçin et al. 2009) but increased liver GPx activity and reduced liver MDA activity in *O. niloticus* exposed to silver nanoparticles (Veisi et al. 2021). These findings reveal a profound interaction between the effects of the SD and ML doses on the antioxidant capacity of *S. aurata* and suggest that administering ML in a dose-density-dependent manner in intensive groundwater aquaculture of *S. aurata* can enhance the antioxidant enzyme system as well as the expression of innate immune markers.

The higher levels of antioxidants and innate immune markers were linked to the role of exogenous ML (1) in encouraging the production of endogenous ML, which is found in large amounts in fish’s digestive tract and helps control eating and digestive functions by acting as an antioxidant (Velarde et al. 2009; Lv et al. 2024); (2) upgrading gut microbes and promoting certain genera that may enhance the animal’s immune function (Mardones et al. 2022; Lv et al. 2024); (3) ML may regulate antioxidant activity via ML receptors and protect against lipid peroxidation by directly removing RO or activating antioxidant enzymes and thus reducing MDA activity (Ghodrati Azadi et al. 2013; Veisi et al. 2021); and (4) since endogenous ML is very sensitive to changes in the environment, like chasing and high SD, which may hinder its synthesis, exogenous ML may boost this synthesis and maintain a stable redox state within cells (Sánchez-Vázquez et al. 2019; Li et al. 2023).

### Stress indicators

There are two neuroendocrine axes in fish that control stress reactions. These are the hypothalamus-pituitary-internal (HPI) axis and the brain sympathetic-chromaffin axis. Stressors activate these axes, which in turn cause elevated stress markers in fish serum, including COR, GLU, and lactate (Sánchez-Vázquez et al. 2019; Oyarzún-Salazar et al. 2022). According to earlier research, ML can help fish cope with a variety of stressors (López-Patiño et al. 2013; Conde-Sieira et al. 2014; Gesto et al. 2016; Moniruzzaman et al. 2020; Drąg-Kozak et al. 2021; Oyarzún-Salazar et al. 2022). In line with previous findings, our results revealed that the serum COR and GLU levels in all ML-treated fish, and serum CRE level in the SD_50_ML_25_ group, were significantly decreased, compared to the control groups, and the effect was dose-density-dependent. One possible explanation for ML’s capacity to reduce stress in teleost fish is the modulation of neuroendocrine responses within the HPI axis (Sánchez-Vázquez et al. 2019) and the generation of an idle, sleeping-like state in fish (Oyarzún-Salazar et al. 2022). Moreover, it has been documented that exogenous ML can improve the oxidative status of fish brains under permethrin stress, stimulate the synthesis of antioxidant proteins through ML receptor proteins, and modulate the expression of heat shock proteins that encourage the brain to become stress-tolerant (Moniruzzaman et al. 2020). Furthermore, ML may also interact with serotonin to control how it works and act instantly on adrenal tissue to change the release of glucocorticoids, just like it does in mammals (Gesto et al. 2016; Haduch et al. 2016).

### Proinflammatory cytokine IL-1β

The interleukin-1 family comprises proinflammatory cytokines that are recruited by macrophages, neutrophils, and lymphocytes (Yusuf et al. 2020) and play an influential role in innate immunity (Sakai et al. 2021). Activated macrophages primarily release IL-1β and activate various proinflammatory transcription elements in diverse target cells (Zhu et al. 2013; Lv et al. 2024). Here, dietary ML supplementation upregulated IL-1β levels in all the treated fish compared to those fed a control diet under both densities. In agreement with these findings, *S. aurata* injected with ML upregulated the levels of IL-1β, along with other immune markers such as interferon-regulatory factor-1, one and three days after injection (Cuesta et al. 2008; Soliman et al. 2025). Likewise, earlier studies have shown that exogenous ML strengthens fish’s immune systems by enhancing macrophage phagocytic activity, cell-mediated cytotoxicity, and the expression of different cytokines, including IL-1β, as well as T- and B-cell markers (Moniruzzaman et al. 2020; Acharyya et al. 2021).

### Histopathology

Fish performance, disease resilience, stress forbearance, and feed efficacy are all influenced by how well their inner organs grow, specifically their gastrointestinal tract and livers (Elhetawy et al. 2024; Lv et al. 2024). The present investigation revealed that adding ML to the *S. aurata* diet had a considerable positive impact on the liver and intestinal tract in all groups. Liver tissues of *S. aurata* treated with ML under SD_100_ conditions demonstrated normal hepatic and pancreatic parts compared with those fed a control diet, which exhibited marked hepatic vacuolization and fatty degenerative changes. Furthermore, the inclusion of ML in the diet enhanced the digestive system, as evidenced by increased intestinal villi length and goblet cell numbers in all supplemented fish compared with those in the control groups (SD_50_ML_0_ and SD_100_ML_0_). These findings align with several earlier investigations (Amri et al. 2020; Miao et al. 2023; Lv et al. 2024; Ye et al. 2024). Previous research shows that adding ML to the diet greatly decreased the size and number of hepatic fat vacuoles in *S. aurata* fingerlings (Amri et al. 2020), improved the morphology and structure of the *M. albus* intestines (Lv et al. 2024), protected the liver and intestinal barrier of common carp from lead-induced toxicity, and enhanced permeability (Miao et al. 2023), and promoted the liver morphology and structure in *P. vannamei* fed with 82.7 mg/kg ML (Ye et al. 2024). This hepatoprotective role of ML can be attributed to its multiple roles in various biological phenomena, including (1) lipid metabolism and the regulation of AST and ALT activity (Amri et al. 2020); (2) boosting the amount of fatty acids with superior oxidative interpretation in hepatic tissue, which facilitates lipid mobilization and increases the effectiveness of the oxidative energy supply (Wu et al. 2022; Ye et al. 2024); (3) declining lipid deposits by promoting the oxidative catabolism of lipids through increasing carnitine palmitoyl transferase 1 activity and impeding fatty acid synthase and acetyl-CoA carboxylase activity (Guo et al. 2013; Qin et al. 2022); and (4) diminishing excessive lipopolysaccharide synthesis and suppressing the transport of lipopolysaccharides derived from blood microbes, alleviating lysosomal imbalance and promoting AMP-activated protein kinase (AMPK) phosphorylation, thus alleviating hepatic cell death and protecting against induced toxicity (Rui et al. 2021; Miao et al. 2023). Furthermore, Lv et al. (2024) attributed the improved gut histomorphology of *M. albus* fed ML-augmented diets to the role of ML in regulating the diversity, quantity and structure of the intestinal flora. Since the morphological characteristics of mucous membranes, including villus height and length alongside goblet cell integers, are generally used to estimate gastrointestinal function, the beneficial effect of ML may involve the rehabilitation of the intestinal barrier and play a central role in reducing stress-induced intestinal inflammation (Miao et al. 2023; Lv et al. 2024). The healthier gut and liver tissues seen in fish fed ML, coupled with better growth performance, higher fish welfare and lower stress indicators, support the long-term administration of dietary ML in intensive aquaculture of *S. aurata* in stressful environments and groundwater conditions.

## Conclusion

The outcomes of this investigation uncovered that dietary ML supplementation improved fish performance in a dose- and density-dependent manner. Fish receiving different doses of ML performed better at both densities in terms of growth, physiology, and histopathology compared with those fed the control diet. Fish fed 50 mg/kg ML under SD_100_ conditions (SD_100_ML_50_ group) recorded higher growth parameters (FW, WG, SGR, and FCR) and carcass protein content than all other fish groups except those fed 25 mg/kg ML under SD_50_ conditions (SD_50_ML_25_ group). Furthermore, both the SD_50_ML_25_ and SD_100_ML_50_ groups exhibited higher GH and IGF1 values than all groups tested. ML supplementation also alleviated density stress and enhanced fish welfare, with decreased COR and healthier intestines and livers. This is supported by the fact that there were significant decreases in TAN, NH_3_, and NO_2_ levels in the culture water of these groups. This study concludes that melatonin is beneficial for reducing stress levels, enhancing fish health, promoting growth and nutrition, and augmenting survival rates. It also reduces cannibalism rates, particularly in conditions of higher fish density. It was determined that an increase in fish density enhances the benefits of melatonin content rising from 25 to 50 mg/kg of feed for fish. In contrast, at low fish density, elevating melatonin concentration from 25 to 50 mg/kg feed is deemed to provide no further advantage. The findings of this study possess practical applicability and are expected to generate a favorable economic impact, particularly by enhancing survival rates, growth, and reducing FCR. The incorporation of melatonin into sea bream diets necessitates further investigative research. Further research directions may include identifying optimal feeding times for ML-enriched diets, the frequency of feedings, and the correlation between melatonin doses and fish size. The varied effects of ML-enriched diets on fish raised in cages vs. those in earthen ponds with algal presence, along with the influence of light intensity and other significant parameters, require further research. Further research is vital to gain a full understanding of the role of dietary ML supplementation in influencing the performance and welfare of intensively farmed fish under harsh farming conditions and the interactions between the exogenous ML dose and SD level in various marine species in the context of groundwater use for mariculture development in new areas.

## Supplementary Information

Below is the link to the electronic supplementary material.

Supplementary Material 1 (DOCX 129 KB)

## Data Availability

Data available upon request from the corresponding author.

## Declarations

### Animal welfare statement

This research work was performed per the guidelines set forth by the NIOF Committee for Institutional Care of Aquatic Organisms and Experimental Animals (NIOF-IACUC), Egypt, and approved under the approval number N0 NIOF-AQ1-F-23-M-049.
