# Supplementary material for: Melatonin supplementation alleviates stocking-density stress and enhances growth, immune, and physiological performance of Sparus aurata cultured in groundwater-based systems
Source: Vet Res Commun. 2025 Dec 25;50(2):84. doi: 10.1007/s11259-025-10992-6 (PMC12740964; doi:10.1007/s11259-025-10992-6)
Supplement: Supplementary file 1 — Supplementary Material 1 (DOCX 129 KB) [file 11259_2025_10992_MOESM1_ESM.docx]

**Table 1**. Tests of Normality using Kolmogorov-Smirnov and Shapiro-Wilk tests.

| **Tests of Normality** | | | | | | |
| --- | --- | --- | --- | --- | --- | --- |
|  | Kolmogorov-Smirnov^a^ | | | Shapiro-Wilk | | |
|  | Statistic | df | Sig. | Statistic | df | Sig. |
| Final Weight, gm/fish | .125 | 18 | .200^*^ | .936 | 18 | .249 |
| Gain, gm/fish | .118 | 18 | .200^*^ | .942 | 18 | .311 |
| ADG, gm/fish/day | .118 | 18 | .200^*^ | .942 | 18 | .311 |
| SGR, %/fish/day | .167 | 18 | .197 | .937 | 18 | .258 |
| Relative growth rate, % | .151 | 18 | .200^*^ | .948 | 18 | .392 |
| Survival, % | .119 | 18 | .200^*^ | .953 | 18 | .472 |
| Feed intake, g/fish | .189 | 18 | .087 | .930 | 18 | .194 |
| FCR | .140 | 18 | .200^*^ | .933 | 18 | .218 |
| PER, gm | .126 | 18 | .200^*^ | .945 | 18 | .353 |
| PPV, % | .172 | 18 | .167 | .943 | 18 | .332 |
| Energy gain, Kcal | .181 | 18 | .123 | .940 | 18 | .286 |
| Energy utilization, % | .106 | 18 | .200^*^ | .960 | 18 | .602 |
| Dry matter, % | .144 | 18 | .200^*^ | .981 | 18 | .963 |
| Protein, % | .126 | 18 | .200^*^ | .963 | 18 | .666 |
| Ether extract, % | .103 | 18 | .200^*^ | .962 | 18 | .634 |
| Ash, % | .126 | 18 | .200^*^ | .971 | 18 | .809 |
| GLUCOSE | .130 | 18 | .200^*^ | .911 | 18 | .091 |
| CHOLEST | .145 | 18 | .200^*^ | .924 | 18 | .149 |
| TRIGLY | .136 | 18 | .200^*^ | .971 | 18 | .814 |
| HDL | .146 | 18 | .200^*^ | .925 | 18 | .158 |
| LDL | .097 | 18 | .200^*^ | .965 | 18 | .698 |
| AMMON | .161 | 18 | .200^*^ | .937 | 18 | .261 |
| UREA | .143 | 18 | .200^*^ | .970 | 18 | .800 |
| CREAT | .132 | 18 | .200^*^ | .937 | 18 | .255 |
| T.PROT | .131 | 18 | .200^*^ | .945 | 18 | .357 |
| Globulin | .155 | 18 | .200^*^ | .961 | 18 | .628 |
| AMYLASE | .123 | 18 | .200^*^ | .981 | 18 | .963 |
| LIPASE | .129 | 18 | .200^*^ | .954 | 18 | .500 |
| LACTATE | .118 | 18 | .200^*^ | .960 | 18 | .610 |
| C3 | .085 | 18 | .200^*^ | .980 | 18 | .948 |
| C4 | .140 | 18 | .200^*^ | .961 | 18 | .622 |
| SOD | .174 | 18 | .155 | .928 | 18 | .180 |
| CAT | .100 | 18 | .200^*^ | .972 | 18 | .838 |
| MDA | .118 | 18 | .200^*^ | .980 | 18 | .949 |
| GPx | .152 | 18 | .200^*^ | .911 | 18 | .090 |
| CORTISOL | .109 | 18 | .200^*^ | .970 | 18 | .799 |
| IGM | .137 | 18 | .200^*^ | .964 | 18 | .674 |
| IGF1 | .114 | 18 | .200^*^ | .976 | 18 | .896 |
| GH | .146 | 18 | .200^*^ | .937 | 18 | .262 |
| IL17B | .156 | 18 | .200^*^ | .950 | 18 | .418 |
|  |  |  |  |  |  |  |
| *. This is a lower bound of the true significance. | | | | | | |
| a. Lilliefors Significance Correction | | | | | | |

**Table 2.** The data of the Eta-squared (**η^2^**) using SPSS.

| \|  \| \| --- \|   **Final Weight, gm/fish** | \| **Directional Measures** \| \| \| \| \| --- \| --- \| --- \| --- \| \|  \| \| \| Value \| \| Nominal by Interval \| Eta \| Treatment Dependent \| 1.000 \| \| Final Weight, gm/fish Dependent \| .994 \| |
| --- | --- | --- | --- | --- | --- | --- | --- | --- | --- | --- | --- | --- | --- | --- | --- | --- |
| **Gain, gm/fish** | \| **Directional Measures** \| \| \| \| \| --- \| --- \| --- \| --- \| \|  \| \| \| Value \| \| Nominal by Interval \| Eta \| Treatment Dependent \| 1.000 \| \| Gain, gm/fish Dependent \| .988 \| |
| ADG, gm/fish/day | \| **Directional Measures** \| \| \| \| \| --- \| --- \| --- \| --- \| \|  \| \| \| Value \| \| Nominal by Interval \| Eta \| Treatment Dependent \| 1.000 \| \| ADG, gm/fish/day Dependent \| .988 \| |
| SGR, %/fish/day | \| **Directional Measures** \| \| \| \| \| --- \| --- \| --- \| --- \| \|  \| \| \| Value \| \| Nominal by Interval \| Eta \| Treatment Dependent \| 1.000 \| \| SGR, %/fish/day Dependent \| .973 \| |
| Relative growth rate, % | \| **Directional Measures** \| \| \| \| \| --- \| --- \| --- \| --- \| \|  \| \| \| Value \| \| Nominal by Interval \| Eta \| Treatment Dependent \| 1.000 \| \| Relative growth rate, % Dependent \| .971 \| |
| Survival, % | \| **Directional Measures** \| \| \| \| \| --- \| --- \| --- \| --- \| \|  \| \| \| Value \| \| Nominal by Interval \| Eta \| Treatment Dependent \| .636 \| \| Survival, % Dependent \| .946 \| |
| Feed intake, g/fish | \| **Directional Measures** \| \| \| \| \| --- \| --- \| --- \| --- \| \|  \| \| \| Value \| \| Nominal by Interval \| Eta \| Treatment Dependent \| 1.000 \| \| Feed intake, g/fish Dependent \| .960 \| |
| FCR | \| **Directional Measures** \| \| \| \| \| --- \| --- \| --- \| --- \| \|  \| \| \| Value \| \| Nominal by Interval \| Eta \| Treatment Dependent \| .900 \| \| FCR Dependent \| .975 \| |
| PER | \| **Directional Measures** \| \| \| \| \| --- \| --- \| --- \| --- \| \|  \| \| \| Value \| \| Nominal by Interval \| Eta \| Treatment Dependent \| .871 \| \| PER, gm Dependent \| .970 \| |
| PPV, % | \| **Directional Measures** \| \| \| \| \| --- \| --- \| --- \| --- \| \|  \| \| \| Value \| \| Nominal by Interval \| Eta \| Treatment Dependent \| 1.000 \| \| PPV, % Dependent \| .967 \| |
| Energy gain, Kcal | \| **Directional Measures** \| \| \| \| \| --- \| --- \| --- \| --- \| \|  \| \| \| Value \| \| Nominal by Interval \| Eta \| Treatment Dependent \| 1.000 \| \| Energy gain, Kcal Dependent \| .980 \| |
| Energy utilization, % | \| **Directional Measures** \| \| \| \| \| --- \| --- \| --- \| --- \| \|  \| \| \| Value \| \| Nominal by Interval \| Eta \| Treatment Dependent \| 1.000 \| \| Energy utilization, % Dependent \| .913 \| |
| Carcass energy, Kcal/100gm | \| **Directional Measures** \| \| \| \| \| --- \| --- \| --- \| --- \| \|  \| \| \| Value \| \| Nominal by Interval \| Eta \| Treatment Dependent \| 1.000 \| \| Carcass energy, Kcal/100gm Dependent \| .809 \| |
| Dry matter, % | \| **Directional Measures** \| \| \| \| \| --- \| --- \| --- \| --- \| \|  \| \| \| Value \| \| Nominal by Interval \| Eta \| Treatment Dependent \| .995 \| \| Dry matter, % Dependent \| .844 \| |
| Protein, % | \| **Directional Measures** \| \| \| \| \| --- \| --- \| --- \| --- \| \|  \| \| \| Value \| \| Nominal by Interval \| Eta \| Treatment Dependent \| 1.000 \| \| Protein, % Dependent \| .874 \| |
| Ether extract, % | \| **Directional Measures** \| \| \| \| \| --- \| --- \| --- \| --- \| \|  \| \| \| Value \| \| Nominal by Interval \| Eta \| Treatment Dependent \| 1.000 \| \| Ether extract, % Dependent \| .890 \| |
| Ash, % | \| **Directional Measures** \| \| \| \| \| --- \| --- \| --- \| --- \| \|  \| \| \| Value \| \| Nominal by Interval \| Eta \| Treatment Dependent \| .956 \| \| Ash, % Dependent \| .464 \| |
| GLUCOSE | \| **Directional Measures** \| \| \| \| \| --- \| --- \| --- \| --- \| \|  \| \| \| Value \| \| Nominal by Interval \| Eta \| Treatment Dependent \| 1.000 \| \| GLUCOSE Dependent \| .989 \| |
| CHOLEST | \| **Directional Measures** \| \| \| \| \| --- \| --- \| --- \| --- \| \|  \| \| \| Value \| \| Nominal by Interval \| Eta \| Treatment Dependent \| 1.000 \| \| CHOLEST Dependent \| .984 \| |
| TRIGLY | \| **Directional Measures** \| \| \| \| \| --- \| --- \| --- \| --- \| \|  \| \| \| Value \| \| Nominal by Interval \| Eta \| Treatment Dependent \| 1.000 \| \| TRIGLY Dependent \| .928 \| |
| HDL | \| **Directional Measures** \| \| \| \| \| --- \| --- \| --- \| --- \| \|  \| \| \| Value \| \| Nominal by Interval \| Eta \| Treatment Dependent \| .889 \| \| HDL Dependent \| .912 \| |
| LDL | \| **Directional Measures** \| \| \| \| \| --- \| --- \| --- \| --- \| \|  \| \| \| Value \| \| Nominal by Interval \| Eta \| Treatment Dependent \| .995 \| \| LDL Dependent \| .987 \| |
| AMMONIA | \| **Directional Measures** \| \| \| \| \| --- \| --- \| --- \| --- \| \|  \| \| \| Value \| \| Nominal by Interval \| Eta \| Treatment Dependent \| .926 \| \| AMMON Dependent \| .917 \| |
| URIC acid | \| **Directional Measures** \| \| \| \| \| --- \| --- \| --- \| --- \| \|  \| \| \| Value \| \| Nominal by Interval \| Eta \| Treatment Dependent \| .995 \| \| URIC Dependent \| .989 \| |
| CREATININE | \| **Directional Measures** \| \| \| \| \| --- \| --- \| --- \| --- \| \|  \| \| \| Value \| \| Nominal by Interval \| Eta \| Treatment Dependent \| .971 \| \| CREAT Dependent \| .715 \| |
| AST | \| **Directional Measures** \| \| \| \| \| --- \| --- \| --- \| --- \| \|  \| \| \| Value \| \| Nominal by Interval \| Eta \| Treatment Dependent \| 1.000 \| \| AST Dependent \| .994 \| |
| ALT | \| **Directional Measures** \| \| \| \| \| --- \| --- \| --- \| --- \| \|  \| \| \| Value \| \| Nominal by Interval \| Eta \| Treatment Dependent \| .931 \| \| ALT Dependent \| .948 \| |
| ALP | \| **Directional Measures** \| \| \| \| \| --- \| --- \| --- \| --- \| \|  \| \| \| Value \| \| Nominal by Interval \| Eta \| Treatment Dependent \| 1.000 \| \| ALP Dependent \| .994 \| |
| T.PROT | \| **Directional Measures** \| \| \| \| \| --- \| --- \| --- \| --- \| \|  \| \| \| Value \| \| Nominal by Interval \| Eta \| Treatment Dependent \| .961 \| \| T.PROT Dependent \| .946 \| |
| Albumin | \| **Directional Measures** \| \| \| \| \| --- \| --- \| --- \| --- \| \|  \| \| \| Value \| \| Nominal by Interval \| Eta \| Treatment Dependent \| .941 \| \| Albumin Dependent \| .946 \| |
| Globulin | \| **Directional Measures** \| \| \| \| \| --- \| --- \| --- \| --- \| \|  \| \| \| Value \| \| Nominal by Interval \| Eta \| Treatment Dependent \| 1.000 \| \| Globulin Dependent \| .881 \| |
| AMYLASE | \| **Directional Measures** \| \| \| \| \| --- \| --- \| --- \| --- \| \|  \| \| \| Value \| \| Nominal by Interval \| Eta \| Treatment Dependent \| .773 \| \| AMYLASE Dependent \| .581 \| |
| LIPASE | \| **Directional Measures** \| \| \| \| \| --- \| --- \| --- \| --- \| \|  \| \| \| Value \| \| Nominal by Interval \| Eta \| Treatment Dependent \| .838 \| \| LIPASE Dependent \| .871 \| |
| LACTATE | \| **Directional Measures** \| \| \| \| \| --- \| --- \| --- \| --- \| \|  \| \| \| Value \| \| Nominal by Interval \| Eta \| Treatment Dependent \| .936 \| \| LACTATE Dependent \| .955 \| |
| C3 | \| **Directional Measures** \| \| \| \| \| --- \| --- \| --- \| --- \| \|  \| \| \| Value \| \| Nominal by Interval \| Eta \| Treatment Dependent \| .971 \| \| C3 Dependent \| .856 \| |
| C4 | \| **Directional Measures** \| \| \| \| \| --- \| --- \| --- \| --- \| \|  \| \| \| Value \| \| Nominal by Interval \| Eta \| Treatment Dependent \| 1.000 \| \| C4 Dependent \| .982 \| |
| SOD | \| **Directional Measures** \| \| \| \| \| --- \| --- \| --- \| --- \| \|  \| \| \| Value \| \| Nominal by Interval \| Eta \| Treatment Dependent \| .884 \| \| SOD Dependent \| .944 \| |
| CAT | \| **Directional Measures** \| \| \| \| \| --- \| --- \| --- \| --- \| \|  \| \| \| Value \| \| Nominal by Interval \| Eta \| Treatment Dependent \| .951 \| \| CAT Dependent \| .919 \| |
| MDA | \| **Directional Measures** \| \| \| \| \| --- \| --- \| --- \| --- \| \|  \| \| \| Value \| \| Nominal by Interval \| Eta \| Treatment Dependent \| .995 \| \| MDA Dependent \| .902 \| |
| GPx | \| **Directional Measures** \| \| \| \| \| --- \| --- \| --- \| --- \| \|  \| \| \| Value \| \| Nominal by Interval \| Eta \| Treatment Dependent \| .976 \| \| GPx Dependent \| .960 \| |
| CORTISOL | \| **Directional Measures** \| \| \| \| \| --- \| --- \| --- \| --- \| \|  \| \| \| Value \| \| Nominal by Interval \| Eta \| Treatment Dependent \| 1.000 \| \| CORTISOL Dependent \| .963 \| |
| IgM | \| **Directional Measures** \| \| \| \| \| --- \| --- \| --- \| --- \| \|  \| \| \| Value \| \| Nominal by Interval \| Eta \| Treatment Dependent \| .976 \| \| IGM Dependent \| .849 \| |
| IGF1 | \| **Directional Measures** \| \| \| \| \| --- \| --- \| --- \| --- \| \|  \| \| \| Value \| \| Nominal by Interval \| Eta \| Treatment Dependent \| .921 \| \| IGF1 Dependent \| .619 \| |
| GH | \| **Directional Measures** \| \| \| \| \| --- \| --- \| --- \| --- \| \|  \| \| \| Value \| \| Nominal by Interval \| Eta \| Treatment Dependent \| .676 \| \| HGH Dependent \| .896 \| |
| IL17B | \| **Directional Measures** \| \| \| \| \| --- \| --- \| --- \| --- \| \|  \| \| \| Value \| \| Nominal by Interval \| Eta \| Treatment Dependent \| .900 \| \| IL17B Dependent \| .867 \| |

**Table 3.** MANOVA data, F-values, mean squares, df, p-values, and Partial Eta square of the statistical results.

| **Tests of Between-Subjects Effects** | | | | | | | | | |
| --- | --- | --- | --- | --- | --- | --- | --- | --- | --- |
| Source | Dependent Variable | Type III Sum of Squares | df | Mean Square | F | Sig. | Partial Eta Squared | Noncent. Parameter | Observed Power^bc^ |
| Corrected Model | Final Weight, gm/fish | 194.587^a^ | 5 | 38.917 | 195.156 | .000 | .988 | 975.781 | 1.000 |
|  | Gain, gm/fish | 195.355^b^ | 5 | 39.071 | 96.429 | .000 | .976 | 482.147 | 1.000 |
|  | ADG, gm/fish/day | .024^c^ | 5 | .005 | 96.426 | .000 | .976 | 482.130 | 1.000 |
|  | SGR, %/fish/day | .141^d^ | 5 | .028 | 43.357 | .000 | .948 | 216.783 | 1.000 |
|  | Relative growth rate, % | 7261.667^e^ | 5 | 1452.333 | 40.117 | .000 | .944 | 200.585 | 1.000 |
|  | Survival, % | 272.000^f^ | 5 | 54.400 | 20.400 | .000 | .895 | 102.000 | 1.000 |
|  | Feed intake, g/fish | 116.108^g^ | 5 | 23.222 | 27.931 | .000 | .921 | 139.656 | 1.000 |
|  | FCR | .241^h^ | 5 | .048 | 45.584 | .000 | .950 | 227.921 | 1.000 |
|  | PER, gm | .200^i^ | 5 | .040 | 38.705 | .000 | .942 | 193.527 | 1.000 |
|  | PPV, % | 260.390^j^ | 5 | 52.078 | 34.057 | .000 | .934 | 170.284 | 1.000 |
|  | Energy gain, Kcal | 1150.985^k^ | 5 | 230.197 | 58.855 | .000 | .961 | 294.273 | 1.000 |
|  | Energy utilization, % | 61.816^l^ | 5 | 12.363 | 12.000 | .000 | .833 | 60.002 | .999 |
|  | Carcass energy, Kcal/100gm | 969.333^m^ | 5 | 193.867 | 4.559 | .015 | .655 | 22.794 | .856 |
|  | Dry matter, % | 11.573^n^ | 5 | 2.315 | 5.957 | .005 | .713 | 29.786 | .940 |
|  | Protein, % | 182.623^o^ | 5 | 36.525 | 7.789 | .002 | .764 | 38.947 | .983 |
|  | Ether extract, % | 198.239^p^ | 5 | 39.648 | 9.174 | .001 | .793 | 45.871 | .994 |
|  | Ash, % | 5.384^q^ | 5 | 1.077 | .658 | .662 | .215 | 3.288 | .169 |
|  | GLUCOSE | 5665.000^r^ | 5 | 1133.000 | 106.219 | .000 | .978 | 531.094 | 1.000 |
|  | CHOLEST | 21064.000^s^ | 5 | 4212.800 | 72.219 | .000 | .968 | 361.097 | 1.000 |
|  | TRIGLY | 5862.000^t^ | 5 | 1172.400 | 14.967 | .000 | .862 | 74.834 | 1.000 |
|  | HDL | 459.000^u^ | 5 | 91.800 | 11.845 | .000 | .832 | 59.226 | .999 |
|  | LDL | 4587.000^v^ | 5 | 917.400 | 88.070 | .000 | .973 | 440.352 | 1.000 |
|  | AMMON | 476.125^w^ | 5 | 95.225 | 12.768 | .000 | .842 | 63.838 | 1.000 |
|  | UREA | 167.500^x^ | 5 | 33.500 | 13.862 | .000 | .852 | 69.310 | 1.000 |
|  | URIC | 2.121^y^ | 5 | .424 | 109.344 | .000 | .979 | 546.719 | 1.000 |
|  | CREAT | .079^z^ | 5 | .016 | 2.514 | .089 | .512 | 12.571 | .575 |
|  | AST | 22607.625^aa^ | 5 | 4521.525 | 192.065 | .000 | .988 | 960.324 | 1.000 |
|  | ALT | 556.000^ab^ | 5 | 111.200 | 21.181 | .000 | .898 | 105.905 | 1.000 |
|  | ALP | 44340.000^ac^ | 5 | 8868.000 | 204.646 | .000 | .988 | 1023.231 | 1.000 |
|  | T.PROT | 2.372^ad^ | 5 | .474 | 20.588 | .000 | .896 | 102.939 | 1.000 |
|  | Albumin | .334^ae^ | 5 | .067 | 20.457 | .000 | .895 | 102.287 | 1.000 |
|  | Globulin | 1.113^af^ | 5 | .223 | 8.356 | .001 | .777 | 41.780 | .989 |
|  | AMYLASE | 52.000^ag^ | 5 | 10.400 | 1.224 | .356 | .338 | 6.118 | .294 |
|  | LIPASE | 223.500^ah^ | 5 | 44.700 | 7.555 | .002 | .759 | 37.775 | .980 |
|  | LACTATE | 1077.610^ai^ | 5 | 215.522 | 24.629 | .000 | .911 | 123.144 | 1.000 |
|  | C3 | 488.125^aj^ | 5 | 97.625 | 6.600 | .004 | .733 | 33.000 | .961 |
|  | C4 | 10.773^ak^ | 5 | 2.155 | 63.784 | .000 | .964 | 318.919 | 1.000 |
|  | SOD | .219^al^ | 5 | .044 | 19.773 | .000 | .892 | 98.865 | 1.000 |
|  | CAT | 7214.500^am^ | 5 | 1442.900 | 13.127 | .000 | .845 | 65.636 | 1.000 |
|  | MDA | .232^an^ | 5 | .046 | 10.420 | .000 | .813 | 52.101 | .998 |
|  | GPx | 1.561^ao^ | 5 | .312 | 28.047 | .000 | .921 | 140.236 | 1.000 |
|  | CORTISOL | 283.706^ap^ | 5 | 56.741 | 30.554 | .000 | .927 | 152.770 | 1.000 |
|  | IGM | 18.381^aq^ | 5 | 3.676 | 6.218 | .005 | .722 | 31.089 | .949 |
|  | IGF1 | 1015.000^ar^ | 5 | 203.000 | 1.487 | .265 | .383 | 7.436 | .355 |
|  | GH | .136^as^ | 5 | .027 | 9.814 | .001 | .804 | 49.069 | .996 |
|  | IL17B | 1381.000^at^ | 5 | 276.200 | 7.284 | .002 | .752 | 36.422 | .975 |
|  | Temperature, 0c | .015^aw^ | 5 | .003 | .008 | 1.000 | .003 | .041 | .051 |
|  | pH | .002^ax^ | 5 | .000 | .239 | .937 | .091 | 1.196 | .088 |
|  | Dissolved oxygen, ppm | .274^ay^ | 5 | .055 | 3.334 | .041 | .581 | 16.671 | .715 |
|  | TAN, ppm | .197^az^ | 5 | .039 | 21.506 | .000 | .900 | 107.530 | 1.000 |
|  | NH3, ppb | 800.578^ba^ | 5 | 160.116 | 10.235 | .001 | .810 | 51.175 | .997 |
|  | NO2, ppb | 4900.813^bb^ | 5 | 980.163 | 18.587 | .000 | .886 | 92.937 | 1.000 |
| Intercept | Final Weight, gm/fish | 32359.680 | 1 | 32359.680 | 162271.692 | .000 | 1.000 | 162271.692 | 1.000 |
|  | Gain, gm/fish | 12112.942 | 1 | 12112.942 | 29895.377 | .000 | 1.000 | 29895.377 | 1.000 |
|  | ADG, gm/fish/day | 1.495 | 1 | 1.495 | 29896.856 | .000 | 1.000 | 29896.856 | 1.000 |
|  | SGR, %/fish/day | 19.770 | 1 | 19.770 | 30415.465 | .000 | 1.000 | 30415.465 | 1.000 |
|  | Relative growth rate, % | 1195130.148 | 1 | 1195130.148 | 33012.375 | .000 | 1.000 | 33012.375 | 1.000 |
|  | Survival, % | 150152.000 | 1 | 150152.000 | 56307.000 | .000 | 1.000 | 56307.000 | 1.000 |
|  | Feed intake, g/fish | 30782.594 | 1 | 30782.594 | 37025.753 | .000 | 1.000 | 37025.753 | 1.000 |
|  | FCR | 46.561 | 1 | 46.561 | 44110.658 | .000 | 1.000 | 44110.658 | 1.000 |
|  | PER, gm | 43.307 | 1 | 43.307 | 41910.022 | .000 | 1.000 | 41910.022 | 1.000 |
|  | PPV, % | 20138.907 | 1 | 20138.907 | 13170.001 | .000 | .999 | 13170.001 | 1.000 |
|  | Energy gain, Kcal | 86406.966 | 1 | 86406.966 | 22091.736 | .000 | .999 | 22091.736 | 1.000 |
|  | Energy utilization, % | 18720.889 | 1 | 18720.889 | 18171.646 | .000 | .999 | 18171.646 | 1.000 |
|  | Carcass energy, Kcal/100gm | 6596450.527 | 1 | 6596450.527 | 155117.165 | .000 | 1.000 | 155117.165 | 1.000 |
|  | Dry matter, % | 20822.963 | 1 | 20822.963 | 53592.995 | .000 | 1.000 | 53592.995 | 1.000 |
|  | Protein, % | 47455.616 | 1 | 47455.616 | 10120.566 | .000 | .999 | 10120.566 | 1.000 |
|  | Ether extract, % | 20433.659 | 1 | 20433.659 | 4728.214 | .000 | .997 | 4728.214 | 1.000 |
|  | Ash, % | 3340.714 | 1 | 3340.714 | 2040.581 | .000 | .994 | 2040.581 | 1.000 |
|  | GLUCOSE | 385442.000 | 1 | 385442.000 | 36135.187 | .000 | 1.000 | 36135.187 | 1.000 |
|  | CHOLEST | 1213682.000 | 1 | 1213682.000 | 20805.977 | .000 | .999 | 20805.977 | 1.000 |
|  | TRIGLY | 2571912.000 | 1 | 2571912.000 | 32832.919 | .000 | 1.000 | 32832.919 | 1.000 |
|  | HDL | 68080.500 | 1 | 68080.500 | 8784.581 | .000 | .999 | 8784.581 | 1.000 |
|  | LDL | 128524.500 | 1 | 128524.500 | 12338.352 | .000 | .999 | 12338.352 | 1.000 |
|  | AMMON | 62835.125 | 1 | 62835.125 | 8424.821 | .000 | .999 | 8424.821 | 1.000 |
|  | UREA | 4232.000 | 1 | 4232.000 | 1751.172 | .000 | .993 | 1751.172 | 1.000 |
|  | URIC | 37.976 | 1 | 37.976 | 9789.632 | .000 | .999 | 9789.632 | 1.000 |
|  | CREAT | 4.898 | 1 | 4.898 | 776.505 | .000 | .985 | 776.505 | 1.000 |
|  | AST | 269011.125 | 1 | 269011.125 | 11427.021 | .000 | .999 | 11427.021 | 1.000 |
|  | ALT | 8064.500 | 1 | 8064.500 | 1536.095 | .000 | .992 | 1536.095 | 1.000 |
|  | ALP | 919368.000 | 1 | 919368.000 | 21216.185 | .000 | .999 | 21216.185 | 1.000 |
|  | T.PROT | 919.991 | 1 | 919.991 | 39920.037 | .000 | 1.000 | 39920.037 | 1.000 |
|  | Albumin | 111.378 | 1 | 111.378 | 34138.793 | .000 | 1.000 | 34138.793 | 1.000 |
|  | Globulin | 391.160 | 1 | 391.160 | 14682.282 | .000 | .999 | 14682.282 | 1.000 |
|  | AMYLASE | 5408.000 | 1 | 5408.000 | 636.235 | .000 | .981 | 636.235 | 1.000 |
|  | LIPASE | 15138.000 | 1 | 15138.000 | 2558.535 | .000 | .995 | 2558.535 | 1.000 |
|  | LACTATE | 70500.125 | 1 | 70500.125 | 8056.390 | .000 | .999 | 8056.390 | 1.000 |
|  | C3 | 26796.125 | 1 | 26796.125 | 1811.569 | .000 | .993 | 1811.569 | 1.000 |
|  | C4 | 416.017 | 1 | 416.017 | 12315.787 | .000 | .999 | 12315.787 | 1.000 |
|  | SOD | 43.524 | 1 | 43.524 | 19635.090 | .000 | .999 | 19635.090 | 1.000 |
|  | CAT | 2957312.000 | 1 | 2957312.000 | 26905.037 | .000 | 1.000 | 26905.037 | 1.000 |
|  | MDA | 43.711 | 1 | 43.711 | 9822.753 | .000 | .999 | 9822.753 | 1.000 |
|  | GPx | 103.464 | 1 | 103.464 | 9296.663 | .000 | .999 | 9296.663 | 1.000 |
|  | CORTISOL | 33398.201 | 1 | 33398.201 | 17984.223 | .000 | .999 | 17984.223 | 1.000 |
|  | IGM | 2467.531 | 1 | 2467.531 | 4173.414 | .000 | .997 | 4173.414 | 1.000 |
|  | IGF1 | 708050.000 | 1 | 708050.000 | 5187.179 | .000 | .998 | 5187.179 | 1.000 |
|  | GH | 26.828 | 1 | 26.828 | 9682.218 | .000 | .999 | 9682.218 | 1.000 |
|  | IL17B | 770040.500 | 1 | 770040.500 | 20308.760 | .000 | .999 | 20308.760 | 1.000 |
|  | Temperature, 0c | 9871.062 | 1 | 9871.062 | 27675.012 | .000 | 1.000 | 27675.012 | 1.000 |
|  | pH | 1201.317 | 1 | 1201.317 | 847988.333 | .000 | 1.000 | 847988.333 | 1.000 |
|  | Dissolved oxygen, ppm | 754.143 | 1 | 754.143 | 45813.635 | .000 | 1.000 | 45813.635 | 1.000 |
|  | TAN, ppm | 3.595 | 1 | 3.595 | 1966.985 | .000 | .994 | 1966.985 | 1.000 |
|  | NH3, ppb | 12912.245 | 1 | 12912.245 | 825.386 | .000 | .986 | 825.386 | 1.000 |
|  | NO2, ppb | 73011.668 | 1 | 73011.668 | 1384.562 | .000 | .991 | 1384.562 | 1.000 |
| Density | Final Weight, gm/fish | 18.932 | 1 | 18.932 | 94.936 | .000 | .888 | 94.936 | 1.000 |
|  | Gain, gm/fish | 18.891 | 1 | 18.891 | 46.623 | .000 | .795 | 46.623 | 1.000 |
|  | ADG, gm/fish/day | .002 | 1 | .002 | 46.622 | .000 | .795 | 46.622 | 1.000 |
|  | SGR, %/fish/day | .014 | 1 | .014 | 21.429 | .001 | .641 | 21.429 | .989 |
|  | Relative growth rate, % | 701.376 | 1 | 701.376 | 19.374 | .001 | .618 | 19.374 | .981 |
|  | Survival, % | 22.222 | 1 | 22.222 | 8.333 | .014 | .410 | 8.333 | .755 |
|  | Feed intake, g/fish | 13.261 | 1 | 13.261 | 15.951 | .002 | .571 | 15.951 | .955 |
|  | FCR | .022 | 1 | .022 | 20.889 | .001 | .635 | 20.889 | .987 |
|  | PER, gm | .017 | 1 | .017 | 16.860 | .001 | .584 | 16.860 | .964 |
|  | PPV, % | 40.620 | 1 | 40.620 | 26.564 | .000 | .689 | 26.564 | .997 |
|  | Energy gain, Kcal | 67.116 | 1 | 67.116 | 17.160 | .001 | .588 | 17.160 | .967 |
|  | Energy utilization, % | 1.186 | 1 | 1.186 | 1.151 | .304 | .088 | 1.151 | .167 |
|  | Carcass energy, Kcal/100gm | 156.114 | 1 | 156.114 | 3.671 | .079 | .234 | 3.671 | .422 |
|  | Dry matter, % | .205 | 1 | .205 | .527 | .482 | .042 | .527 | .103 |
|  | Protein, % | 68.562 | 1 | 68.562 | 14.622 | .002 | .549 | 14.622 | .939 |
|  | Ether extract, % | 56.996 | 1 | 56.996 | 13.188 | .003 | .524 | 13.188 | .914 |
|  | Ash, % | .218 | 1 | .218 | .133 | .722 | .011 | .133 | .063 |
|  | GLUCOSE | 1404.500 | 1 | 1404.500 | 131.672 | .000 | .916 | 131.672 | 1.000 |
|  | CHOLEST | 4050.000 | 1 | 4050.000 | 69.429 | .000 | .853 | 69.429 | 1.000 |
|  | TRIGLY | 72.000 | 1 | 72.000 | .919 | .357 | .071 | .919 | .143 |
|  | HDL | 84.500 | 1 | 84.500 | 10.903 | .006 | .476 | 10.903 | .857 |
|  | LDL | 2812.500 | 1 | 2812.500 | 270.000 | .000 | .957 | 270.000 | 1.000 |
|  | AMMON | .125 | 1 | .125 | .017 | .899 | .001 | .017 | .052 |
|  | UREA | 4.500 | 1 | 4.500 | 1.862 | .197 | .134 | 1.862 | .242 |
|  | URIC | .788 | 1 | .788 | 203.011 | .000 | .944 | 203.011 | 1.000 |
|  | CREAT | .007 | 1 | .007 | 1.141 | .306 | .087 | 1.141 | .166 |
|  | AST | 190.125 | 1 | 190.125 | 8.076 | .015 | .402 | 8.076 | .742 |
|  | ALT | 180.500 | 1 | 180.500 | 34.381 | .000 | .741 | 34.381 | 1.000 |
|  | ALP | 144.500 | 1 | 144.500 | 3.335 | .093 | .217 | 3.335 | .390 |
|  | T.PROT | .475 | 1 | .475 | 20.625 | .001 | .632 | 20.625 | .986 |
|  | Albumin | .011 | 1 | .011 | 3.222 | .098 | .212 | 3.222 | .379 |
|  | Globulin | .344 | 1 | .344 | 12.929 | .004 | .519 | 12.929 | .909 |
|  | AMYLASE | 8.000 | 1 | 8.000 | .941 | .351 | .073 | .941 | .145 |
|  | LIPASE | 4.500 | 1 | 4.500 | .761 | .400 | .060 | .761 | .127 |
|  | LACTATE | 351.125 | 1 | 351.125 | 40.125 | .000 | .770 | 40.125 | 1.000 |
|  | C3 | 28.125 | 1 | 28.125 | 1.901 | .193 | .137 | 1.901 | .246 |
|  | C4 | 6.790 | 1 | 6.790 | 201.000 | .000 | .944 | 201.000 | 1.000 |
|  | SOD | .008 | 1 | .008 | 3.812 | .075 | .241 | 3.812 | .435 |
|  | CAT | 1512.500 | 1 | 1512.500 | 13.760 | .003 | .534 | 13.760 | .925 |
|  | MDA | .007 | 1 | .007 | 1.529 | .240 | .113 | 1.529 | .207 |
|  | GPx | .007 | 1 | .007 | .594 | .456 | .047 | .594 | .110 |
|  | CORTISOL | 58.861 | 1 | 58.861 | 31.696 | .000 | .725 | 31.696 | .999 |
|  | IGM | .661 | 1 | .661 | 1.118 | .311 | .085 | 1.118 | .164 |
|  | IGF1 | 338.000 | 1 | 338.000 | 2.476 | .142 | .171 | 2.476 | .305 |
|  | GH | .012 | 1 | .012 | 4.335 | .059 | .265 | 4.335 | .482 |
|  | IL17B | 72.000 | 1 | 72.000 | 1.899 | .193 | .137 | 1.899 | .245 |
|  | Temperature, 0c | .000 | 1 | .000 | .001 | .975 | .000 | .001 | .050 |
|  | pH | .000 | 1 | .000 | .098 | .760 | .008 | .098 | .060 |
|  | Dissolved oxygen, ppm | .243 | 1 | .243 | 14.742 | .002 | .551 | 14.742 | .940 |
|  | TAN, ppm | .154 | 1 | .154 | 84.171 | .000 | .875 | 84.171 | 1.000 |
|  | NH3, ppb | 583.681 | 1 | 583.681 | 37.310 | .000 | .757 | 37.310 | 1.000 |
|  | NO2, ppb | 2272.683 | 1 | 2272.683 | 43.098 | .000 | .782 | 43.098 | 1.000 |
| Melatonin | Final Weight, gm/fish | 135.250 | 2 | 67.625 | 339.113 | .000 | .983 | 678.226 | 1.000 |
|  | Gain, gm/fish | 136.074 | 2 | 68.037 | 167.919 | .000 | .966 | 335.838 | 1.000 |
|  | ADG, gm/fish/day | .017 | 2 | .008 | 167.914 | .000 | .966 | 335.828 | 1.000 |
|  | SGR, %/fish/day | .099 | 2 | .050 | 76.373 | .000 | .927 | 152.745 | 1.000 |
|  | Relative growth rate, % | 5063.880 | 2 | 2531.940 | 69.938 | .000 | .921 | 139.877 | 1.000 |
|  | Survival, % | 201.333 | 2 | 100.667 | 37.750 | .000 | .863 | 75.500 | 1.000 |
|  | Feed intake, g/fish | 89.883 | 2 | 44.941 | 54.056 | .000 | .900 | 108.112 | 1.000 |
|  | FCR | .151 | 2 | .075 | 71.353 | .000 | .922 | 142.705 | 1.000 |
|  | PER, gm | .123 | 2 | .062 | 59.618 | .000 | .909 | 119.237 | 1.000 |
|  | PPV, % | 202.413 | 2 | 101.206 | 66.185 | .000 | .917 | 132.369 | 1.000 |
|  | Energy gain, Kcal | 902.048 | 2 | 451.024 | 115.314 | .000 | .951 | 230.627 | 1.000 |
|  | Energy utilization, % | 48.562 | 2 | 24.281 | 23.568 | .000 | .797 | 47.137 | 1.000 |
|  | Carcass energy, Kcal/100gm | 589.448 | 2 | 294.724 | 6.931 | .010 | .536 | 13.861 | .841 |
|  | Dry matter, % | 1.133 | 2 | .566 | 1.458 | .271 | .195 | 2.916 | .252 |
|  | Protein, % | 24.360 | 2 | 12.180 | 2.598 | .116 | .302 | 5.195 | .419 |
|  | Ether extract, % | 39.756 | 2 | 19.878 | 4.600 | .033 | .434 | 9.199 | .663 |
|  | Ash, % | 3.015 | 2 | 1.507 | .921 | .425 | .133 | 1.842 | .173 |
|  | GLUCOSE | 685.750 | 2 | 342.875 | 32.145 | .000 | .843 | 64.289 | 1.000 |
|  | CHOLEST | 15505.750 | 2 | 7752.875 | 132.906 | .000 | .957 | 265.813 | 1.000 |
|  | TRIGLY | 4467.000 | 2 | 2233.500 | 28.513 | .000 | .826 | 57.026 | 1.000 |
|  | HDL | 279.750 | 2 | 139.875 | 18.048 | .000 | .751 | 36.097 | .998 |
|  | LDL | 1521.750 | 2 | 760.875 | 73.044 | .000 | .924 | 146.088 | 1.000 |
|  | AMMON | 151.750 | 2 | 75.875 | 10.173 | .003 | .629 | 20.346 | .952 |
|  | UREA | 106.750 | 2 | 53.375 | 22.086 | .000 | .786 | 44.172 | 1.000 |
|  | URIC | .178 | 2 | .089 | 22.892 | .000 | .792 | 45.783 | 1.000 |
|  | CREAT | .023 | 2 | .012 | 1.845 | .200 | .235 | 3.690 | .310 |
|  | AST | 42.750 | 2 | 21.375 | .908 | .429 | .131 | 1.816 | .171 |
|  | ALT | 268.750 | 2 | 134.375 | 25.595 | .000 | .810 | 51.190 | 1.000 |
|  | ALP | 855.750 | 2 | 427.875 | 9.874 | .003 | .622 | 19.748 | .946 |
|  | T.PROT | 1.544 | 2 | .772 | 33.508 | .000 | .848 | 67.015 | 1.000 |
|  | Albumin | .187 | 2 | .093 | 28.655 | .000 | .827 | 57.310 | 1.000 |
|  | Globulin | .657 | 2 | .329 | 12.339 | .001 | .673 | 24.678 | .980 |
|  | AMYLASE | 7.000 | 2 | 3.500 | .412 | .671 | .064 | .824 | .102 |
|  | LIPASE | 15.750 | 2 | 7.875 | 1.331 | .301 | .182 | 2.662 | .233 |
|  | LACTATE | 336.018 | 2 | 168.009 | 19.199 | .000 | .762 | 38.398 | .999 |
|  | C3 | 346.750 | 2 | 173.375 | 11.721 | .002 | .661 | 23.442 | .974 |
|  | C4 | 3.879 | 2 | 1.940 | 57.421 | .000 | .905 | 114.843 | 1.000 |
|  | SOD | .136 | 2 | .068 | 30.671 | .000 | .836 | 61.342 | 1.000 |
|  | CAT | 3912.250 | 2 | 1956.125 | 17.796 | .000 | .748 | 35.593 | .998 |
|  | MDA | .210 | 2 | .105 | 23.554 | .000 | .797 | 47.109 | 1.000 |
|  | GPx | 1.237 | 2 | .618 | 55.570 | .000 | .903 | 111.140 | 1.000 |
|  | CORTISOL | 211.328 | 2 | 105.664 | 56.898 | .000 | .905 | 113.795 | 1.000 |
|  | IGM | 7.330 | 2 | 3.665 | 6.199 | .014 | .508 | 12.397 | .796 |
|  | IGF1 | 73.000 | 2 | 36.500 | .267 | .770 | .043 | .535 | .083 |
|  | GH | .056 | 2 | .028 | 10.182 | .003 | .629 | 20.364 | .952 |
|  | IL17B | 219.250 | 2 | 109.625 | 2.891 | .094 | .325 | 5.782 | .460 |
|  | Temperature, 0c | .010 | 2 | .005 | .014 | .986 | .002 | .029 | .052 |
|  | pH | .001 | 2 | .001 | .522 | .606 | .080 | 1.043 | .117 |
|  | Dissolved oxygen, ppm | .029 | 2 | .014 | .867 | .445 | .126 | 1.734 | .165 |
|  | TAN, ppm | .037 | 2 | .019 | 10.253 | .003 | .631 | 20.505 | .953 |
|  | NH3, ppb | 199.243 | 2 | 99.622 | 6.368 | .013 | .515 | 12.736 | .808 |
|  | NO2, ppb | 2000.150 | 2 | 1000.075 | 18.965 | .000 | .760 | 37.930 | .999 |
| Density * Melatonin | Final Weight, gm/fish | 40.406 | 2 | 20.203 | 101.310 | .000 | .944 | 202.619 | 1.000 |
|  | Gain, gm/fish | 40.390 | 2 | 20.195 | 49.843 | .000 | .893 | 99.686 | 1.000 |
|  | ADG, gm/fish/day | .005 | 2 | .002 | 49.840 | .000 | .893 | 99.680 | 1.000 |
|  | SGR, %/fish/day | .028 | 2 | .014 | 21.305 | .000 | .780 | 42.609 | 1.000 |
|  | Relative growth rate, % | 1496.411 | 2 | 748.205 | 20.667 | .000 | .775 | 41.334 | .999 |
|  | Survival, % | 48.444 | 2 | 24.222 | 9.083 | .004 | .602 | 18.167 | .927 |
|  | Feed intake, g/fish | 12.964 | 2 | 6.482 | 7.796 | .007 | .565 | 15.593 | .883 |
|  | FCR | .068 | 2 | .034 | 32.163 | .000 | .843 | 64.326 | 1.000 |
|  | PER, gm | .059 | 2 | .030 | 28.715 | .000 | .827 | 57.430 | 1.000 |
|  | PPV, % | 17.357 | 2 | 8.678 | 5.675 | .018 | .486 | 11.351 | .759 |
|  | Energy gain, Kcal | 181.821 | 2 | 90.910 | 23.243 | .000 | .795 | 46.486 | 1.000 |
|  | Energy utilization, % | 12.068 | 2 | 6.034 | 5.857 | .017 | .494 | 11.714 | .772 |
|  | Carcass energy, Kcal/100gm | 223.771 | 2 | 111.885 | 2.631 | .113 | .305 | 5.262 | .424 |
|  | Dry matter, % | 10.235 | 2 | 5.118 | 13.172 | .001 | .687 | 26.343 | .986 |
|  | Protein, % | 89.701 | 2 | 44.850 | 9.565 | .003 | .615 | 19.130 | .939 |
|  | Ether extract, % | 101.487 | 2 | 50.744 | 11.742 | .001 | .662 | 23.484 | .974 |
|  | Ash, % | 2.151 | 2 | 1.075 | .657 | .536 | .099 | 1.314 | .135 |
|  | GLUCOSE | 3574.750 | 2 | 1787.375 | 167.566 | .000 | .965 | 335.133 | 1.000 |
|  | CHOLEST | 1508.250 | 2 | 754.125 | 12.928 | .001 | .683 | 25.856 | .984 |
|  | TRIGLY | 1323.000 | 2 | 661.500 | 8.445 | .005 | .585 | 16.889 | .907 |
|  | HDL | 94.750 | 2 | 47.375 | 6.113 | .015 | .505 | 12.226 | .791 |
|  | LDL | 252.750 | 2 | 126.375 | 12.132 | .001 | .669 | 24.264 | .978 |
|  | AMMON | 324.250 | 2 | 162.125 | 21.737 | .000 | .784 | 43.475 | 1.000 |
|  | UREA | 56.250 | 2 | 28.125 | 11.638 | .002 | .660 | 23.276 | .973 |
|  | URIC | 1.156 | 2 | .578 | 148.962 | .000 | .961 | 297.925 | 1.000 |
|  | CREAT | .049 | 2 | .024 | 3.870 | .050 | .392 | 7.740 | .584 |
|  | AST | 22374.750 | 2 | 11187.375 | 475.216 | .000 | .988 | 950.432 | 1.000 |
|  | ALT | 106.750 | 2 | 53.375 | 10.167 | .003 | .629 | 20.333 | .951 |
|  | ALP | 43339.750 | 2 | 21669.875 | 500.074 | .000 | .988 | 1000.148 | 1.000 |
|  | T.PROT | .353 | 2 | .176 | 7.649 | .007 | .560 | 15.299 | .876 |
|  | Albumin | .136 | 2 | .068 | 20.877 | .000 | .777 | 41.755 | 1.000 |
|  | Globulin | .111 | 2 | .056 | 2.086 | .167 | .258 | 4.173 | .345 |
|  | AMYLASE | 37.000 | 2 | 18.500 | 2.176 | .156 | .266 | 4.353 | .359 |
|  | LIPASE | 203.250 | 2 | 101.625 | 17.176 | .000 | .741 | 34.352 | .997 |
|  | LACTATE | 390.468 | 2 | 195.234 | 22.310 | .000 | .788 | 44.621 | 1.000 |
|  | C3 | 113.250 | 2 | 56.625 | 3.828 | .052 | .390 | 7.656 | .579 |
|  | C4 | .104 | 2 | .052 | 1.538 | .254 | .204 | 3.076 | .264 |
|  | SOD | .075 | 2 | .037 | 16.855 | .000 | .737 | 33.711 | .997 |
|  | CAT | 1789.750 | 2 | 894.875 | 8.141 | .006 | .576 | 16.283 | .896 |
|  | MDA | .015 | 2 | .008 | 1.732 | .218 | .224 | 3.463 | .293 |
|  | GPx | .317 | 2 | .159 | 14.251 | .001 | .704 | 28.502 | .991 |
|  | CORTISOL | 13.517 | 2 | 6.759 | 3.639 | .058 | .378 | 7.279 | .556 |
|  | IGM | 10.390 | 2 | 5.195 | 8.786 | .004 | .594 | 17.573 | .918 |
|  | IGF1 | 604.000 | 2 | 302.000 | 2.212 | .152 | .269 | 4.425 | .364 |
|  | GH | .068 | 2 | .034 | 12.185 | .001 | .670 | 24.370 | .978 |
|  | IL17B | 1089.750 | 2 | 544.875 | 14.370 | .001 | .705 | 28.741 | .991 |
|  | Temperature, 0c | .004 | 2 | .002 | .006 | .994 | .001 | .011 | .051 |
|  | pH | 7.778E-5 | 2 | 3.889E-5 | .027 | .973 | .005 | .055 | .053 |
|  | Dissolved oxygen, ppm | .003 | 2 | .002 | .098 | .908 | .016 | .195 | .062 |
|  | TAN, ppm | .005 | 2 | .003 | 1.427 | .278 | .192 | 2.853 | .247 |
|  | NH3, ppb | 17.654 | 2 | 8.827 | .564 | .583 | .086 | 1.129 | .123 |
|  | NO2, ppb | 627.980 | 2 | 313.990 | 5.954 | .016 | .498 | 11.909 | .779 |
| Error | Final Weight, gm/fish | 2.393 | 12 | .199 |  |  |  |  |  |
|  | Gain, gm/fish | 4.862 | 12 | .405 |  |  |  |  |  |
|  | ADG, gm/fish/day | .001 | 12 | 5.002E-5 |  |  |  |  |  |
|  | SGR, %/fish/day | .008 | 12 | .001 |  |  |  |  |  |
|  | Relative growth rate, % | 434.430 | 12 | 36.202 |  |  |  |  |  |
|  | Survival, % | 32.000 | 12 | 2.667 |  |  |  |  |  |
|  | Feed intake, g/fish | 9.977 | 12 | .831 |  |  |  |  |  |
|  | FCR | .013 | 12 | .001 |  |  |  |  |  |
|  | PER, gm | .012 | 12 | .001 |  |  |  |  |  |
|  | PPV, % | 18.350 | 12 | 1.529 |  |  |  |  |  |
|  | Energy gain, Kcal | 46.935 | 12 | 3.911 |  |  |  |  |  |
|  | Energy utilization, % | 12.363 | 12 | 1.030 |  |  |  |  |  |
|  | Carcass energy, Kcal/100gm | 510.307 | 12 | 42.526 |  |  |  |  |  |
|  | Dry matter, % | 4.662 | 12 | .389 |  |  |  |  |  |
|  | Protein, % | 56.268 | 12 | 4.689 |  |  |  |  |  |
|  | Ether extract, % | 51.860 | 12 | 4.322 |  |  |  |  |  |
|  | Ash, % | 19.646 | 12 | 1.637 |  |  |  |  |  |
|  | GLUCOSE | 128.000 | 12 | 10.667 |  |  |  |  |  |
|  | CHOLEST | 700.000 | 12 | 58.333 |  |  |  |  |  |
|  | TRIGLY | 940.000 | 12 | 78.333 |  |  |  |  |  |
|  | HDL | 93.000 | 12 | 7.750 |  |  |  |  |  |
|  | LDL | 125.000 | 12 | 10.417 |  |  |  |  |  |
|  | AMMON | 89.500 | 12 | 7.458 |  |  |  |  |  |
|  | UREA | 29.000 | 12 | 2.417 |  |  |  |  |  |
|  | URIC | .047 | 12 | .004 |  |  |  |  |  |
|  | CREAT | .076 | 12 | .006 |  |  |  |  |  |
|  | AST | 282.500 | 12 | 23.542 |  |  |  |  |  |
|  | ALT | 63.000 | 12 | 5.250 |  |  |  |  |  |
|  | ALP | 520.000 | 12 | 43.333 |  |  |  |  |  |
|  | T.PROT | .277 | 12 | .023 |  |  |  |  |  |
|  | Albumin | .039 | 12 | .003 |  |  |  |  |  |
|  | Globulin | .320 | 12 | .027 |  |  |  |  |  |
|  | AMYLASE | 102.000 | 12 | 8.500 |  |  |  |  |  |
|  | LIPASE | 71.000 | 12 | 5.917 |  |  |  |  |  |
|  | LACTATE | 105.010 | 12 | 8.751 |  |  |  |  |  |
|  | C3 | 177.500 | 12 | 14.792 |  |  |  |  |  |
|  | C4 | .405 | 12 | .034 |  |  |  |  |  |
|  | SOD | .027 | 12 | .002 |  |  |  |  |  |
|  | CAT | 1319.000 | 12 | 109.917 |  |  |  |  |  |
|  | MDA | .053 | 12 | .004 |  |  |  |  |  |
|  | GPx | .134 | 12 | .011 |  |  |  |  |  |
|  | CORTISOL | 22.285 | 12 | 1.857 |  |  |  |  |  |
|  | IGM | 7.095 | 12 | .591 |  |  |  |  |  |
|  | IGF1 | 1638.000 | 12 | 136.500 |  |  |  |  |  |
|  | GH | .033 | 12 | .003 |  |  |  |  |  |
|  | IL17B | 455.000 | 12 | 37.917 |  |  |  |  |  |
|  | Temperature, 0c | 4.280 | 12 | .357 |  |  |  |  |  |
|  | pH | .017 | 12 | .001 |  |  |  |  |  |
|  | Dissolved oxygen, ppm | .198 | 12 | .016 |  |  |  |  |  |
|  | TAN, ppm | .022 | 12 | .002 |  |  |  |  |  |
|  | NH3, ppb | 187.727 | 12 | 15.644 |  |  |  |  |  |
|  | NO2, ppb | 632.792 | 12 | 52.733 |  |  |  |  |  |
| Total | Final Weight, gm/fish | 32556.660 | 18 |  |  |  |  |  |  |
|  | Gain, gm/fish | 12313.160 | 18 |  |  |  |  |  |  |
|  | ADG, gm/fish/day | 1.520 | 18 |  |  |  |  |  |  |
|  | SGR, %/fish/day | 19.919 | 18 |  |  |  |  |  |  |
|  | Relative growth rate, % | 1202826.245 | 18 |  |  |  |  |  |  |
|  | Survival, % | 150456.000 | 18 |  |  |  |  |  |  |
|  | Feed intake, g/fish | 30908.679 | 18 |  |  |  |  |  |  |
|  | FCR | 46.815 | 18 |  |  |  |  |  |  |
|  | PER, gm | 43.519 | 18 |  |  |  |  |  |  |
|  | PPV, % | 20417.646 | 18 |  |  |  |  |  |  |
|  | Energy gain, Kcal | 87604.886 | 18 |  |  |  |  |  |  |
|  | Energy utilization, % | 18795.068 | 18 |  |  |  |  |  |  |
|  | Carcass energy, Kcal/100gm | 6597930.167 | 18 |  |  |  |  |  |  |
|  | Dry matter, % | 20839.198 | 18 |  |  |  |  |  |  |
|  | Protein, % | 47694.508 | 18 |  |  |  |  |  |  |
|  | Ether extract, % | 20683.758 | 18 |  |  |  |  |  |  |
|  | Ash, % | 3365.743 | 18 |  |  |  |  |  |  |
|  | GLUCOSE | 391235.000 | 18 |  |  |  |  |  |  |
|  | CHOLEST | 1235446.000 | 18 |  |  |  |  |  |  |
|  | TRIGLY | 2578714.000 | 18 |  |  |  |  |  |  |
|  | HDL | 68632.500 | 18 |  |  |  |  |  |  |
|  | LDL | 133236.500 | 18 |  |  |  |  |  |  |
|  | AMMON | 63400.750 | 18 |  |  |  |  |  |  |
|  | UREA | 4428.500 | 18 |  |  |  |  |  |  |
|  | URIC | 40.143 | 18 |  |  |  |  |  |  |
|  | CREAT | 5.053 | 18 |  |  |  |  |  |  |
|  | AST | 291901.250 | 18 |  |  |  |  |  |  |
|  | ALT | 8683.500 | 18 |  |  |  |  |  |  |
|  | ALP | 964228.000 | 18 |  |  |  |  |  |  |
|  | T.PROT | 922.639 | 18 |  |  |  |  |  |  |
|  | Albumin | 111.751 | 18 |  |  |  |  |  |  |
|  | Globulin | 392.593 | 18 |  |  |  |  |  |  |
|  | AMYLASE | 5562.000 | 18 |  |  |  |  |  |  |
|  | LIPASE | 15432.500 | 18 |  |  |  |  |  |  |
|  | LACTATE | 71682.745 | 18 |  |  |  |  |  |  |
|  | C3 | 27461.750 | 18 |  |  |  |  |  |  |
|  | C4 | 427.195 | 18 |  |  |  |  |  |  |
|  | SOD | 43.770 | 18 |  |  |  |  |  |  |
|  | CAT | 2965845.500 | 18 |  |  |  |  |  |  |
|  | MDA | 43.996 | 18 |  |  |  |  |  |  |
|  | GPx | 105.158 | 18 |  |  |  |  |  |  |
|  | CORTISOL | 33704.193 | 18 |  |  |  |  |  |  |
|  | IGM | 2493.007 | 18 |  |  |  |  |  |  |
|  | IGF1 | 710703.000 | 18 |  |  |  |  |  |  |
|  | GH | 26.997 | 18 |  |  |  |  |  |  |
|  | IL17B | 771876.500 | 18 |  |  |  |  |  |  |
|  | Temperature, 0c | 9875.356 | 18 |  |  |  |  |  |  |
|  | pH | 1201.336 | 18 |  |  |  |  |  |  |
|  | Dissolved oxygen, ppm | 754.615 | 18 |  |  |  |  |  |  |
|  | TAN, ppm | 3.813 | 18 |  |  |  |  |  |  |
|  | NH3, ppb | 13900.550 | 18 |  |  |  |  |  |  |
|  | NO2, ppb | 78545.274 | 18 |  |  |  |  |  |  |
| Corrected Total | Final Weight, gm/fish | 196.980 | 17 |  |  |  |  |  |  |
|  | Gain, gm/fish | 200.218 | 17 |  |  |  |  |  |  |
|  | ADG, gm/fish/day | .025 | 17 |  |  |  |  |  |  |
|  | SGR, %/fish/day | .149 | 17 |  |  |  |  |  |  |
|  | Relative growth rate, % | 7696.097 | 17 |  |  |  |  |  |  |
|  | Survival, % | 304.000 | 17 |  |  |  |  |  |  |
|  | Feed intake, g/fish | 126.084 | 17 |  |  |  |  |  |  |
|  | FCR | .253 | 17 |  |  |  |  |  |  |
|  | PER, gm | .212 | 17 |  |  |  |  |  |  |
|  | PPV, % | 278.739 | 17 |  |  |  |  |  |  |
|  | Energy gain, Kcal | 1197.920 | 17 |  |  |  |  |  |  |
|  | Energy utilization, % | 74.179 | 17 |  |  |  |  |  |  |
|  | Carcass energy, Kcal/100gm | 1479.640 | 17 |  |  |  |  |  |  |
|  | Dry matter, % | 16.236 | 17 |  |  |  |  |  |  |
|  | Protein, % | 238.892 | 17 |  |  |  |  |  |  |
|  | Ether extract, % | 250.099 | 17 |  |  |  |  |  |  |
|  | Ash, % | 25.029 | 17 |  |  |  |  |  |  |
|  | GLUCOSE | 5793.000 | 17 |  |  |  |  |  |  |
|  | CHOLEST | 21764.000 | 17 |  |  |  |  |  |  |
|  | TRIGLY | 6802.000 | 17 |  |  |  |  |  |  |
|  | HDL | 552.000 | 17 |  |  |  |  |  |  |
|  | LDL | 4712.000 | 17 |  |  |  |  |  |  |
|  | AMMON | 565.625 | 17 |  |  |  |  |  |  |
|  | UREA | 196.500 | 17 |  |  |  |  |  |  |
|  | URIC | 2.167 | 17 |  |  |  |  |  |  |
|  | CREAT | .155 | 17 |  |  |  |  |  |  |
|  | AST | 22890.125 | 17 |  |  |  |  |  |  |
|  | ALT | 619.000 | 17 |  |  |  |  |  |  |
|  | ALP | 44860.000 | 17 |  |  |  |  |  |  |
|  | T.PROT | 2.649 | 17 |  |  |  |  |  |  |
|  | Albumin | .373 | 17 |  |  |  |  |  |  |
|  | Globulin | 1.433 | 17 |  |  |  |  |  |  |
|  | AMYLASE | 154.000 | 17 |  |  |  |  |  |  |
|  | LIPASE | 294.500 | 17 |  |  |  |  |  |  |
|  | LACTATE | 1182.620 | 17 |  |  |  |  |  |  |
|  | C3 | 665.625 | 17 |  |  |  |  |  |  |
|  | C4 | 11.178 | 17 |  |  |  |  |  |  |
|  | SOD | .246 | 17 |  |  |  |  |  |  |
|  | CAT | 8533.500 | 17 |  |  |  |  |  |  |
|  | MDA | .285 | 17 |  |  |  |  |  |  |
|  | GPx | 1.694 | 17 |  |  |  |  |  |  |
|  | CORTISOL | 305.991 | 17 |  |  |  |  |  |  |
|  | IGM | 25.476 | 17 |  |  |  |  |  |  |
|  | IGF1 | 2653.000 | 17 |  |  |  |  |  |  |
|  | GH | .169 | 17 |  |  |  |  |  |  |
|  | IL17B | 1836.000 | 17 |  |  |  |  |  |  |
|  | Temperature, 0c | 4.295 | 17 |  |  |  |  |  |  |
|  | pH | .019 | 17 |  |  |  |  |  |  |
|  | Dissolved oxygen, ppm | .472 | 17 |  |  |  |  |  |  |
|  | TAN, ppm | .218 | 17 |  |  |  |  |  |  |
|  | NH3, ppb | 988.305 | 17 |  |  |  |  |  |  |
|  | NO2, ppb | 5533.605 | 17 |  |  |  |  |  |  |
| a. R Squared = .988 (Adjusted R Squared = .983) | | | | | | | | | |
| b. R Squared = .976 (Adjusted R Squared = .966) | | | | | | | | | |
| c. R Squared = .976 (Adjusted R Squared = .966) | | | | | | | | | |
| d. R Squared = .948 (Adjusted R Squared = .926) | | | | | | | | | |
| e. R Squared = .944 (Adjusted R Squared = .920) | | | | | | | | | |
| f. R Squared = .895 (Adjusted R Squared = .851) | | | | | | | | | |
| g. R Squared = .921 (Adjusted R Squared = .888) | | | | | | | | | |
| h. R Squared = .950 (Adjusted R Squared = .929) | | | | | | | | | |
| i. R Squared = .942 (Adjusted R Squared = .917) | | | | | | | | | |
| j. R Squared = .934 (Adjusted R Squared = .907) | | | | | | | | | |
| k. R Squared = .961 (Adjusted R Squared = .944) | | | | | | | | | |
| l. R Squared = .833 (Adjusted R Squared = .764) | | | | | | | | | |
| m. R Squared = .655 (Adjusted R Squared = .511) | | | | | | | | | |
| n. R Squared = .713 (Adjusted R Squared = .593) | | | | | | | | | |
| o. R Squared = .764 (Adjusted R Squared = .666) | | | | | | | | | |
| p. R Squared = .793 (Adjusted R Squared = .706) | | | | | | | | | |
| q. R Squared = .215 (Adjusted R Squared = -.112) | | | | | | | | | |
| r. R Squared = .978 (Adjusted R Squared = .969) | | | | | | | | | |
| s. R Squared = .968 (Adjusted R Squared = .954) | | | | | | | | | |
| t. R Squared = .862 (Adjusted R Squared = .804) | | | | | | | | | |
| u. R Squared = .832 (Adjusted R Squared = .761) | | | | | | | | | |
| v. R Squared = .973 (Adjusted R Squared = .962) | | | | | | | | | |
| w. R Squared = .842 (Adjusted R Squared = .776) | | | | | | | | | |
| x. R Squared = .852 (Adjusted R Squared = .791) | | | | | | | | | |
| y. R Squared = .979 (Adjusted R Squared = .970) | | | | | | | | | |
| z. R Squared = .512 (Adjusted R Squared = .308) | | | | | | | | | |
| aa. R Squared = .988 (Adjusted R Squared = .983) | | | | | | | | | |
| ab. R Squared = .898 (Adjusted R Squared = .856) | | | | | | | | | |
| ac. R Squared = .988 (Adjusted R Squared = .984) | | | | | | | | | |
| ad. R Squared = .896 (Adjusted R Squared = .852) | | | | | | | | | |
| ae. R Squared = .895 (Adjusted R Squared = .851) | | | | | | | | | |
| af. R Squared = .777 (Adjusted R Squared = .684) | | | | | | | | | |
| ag. R Squared = .338 (Adjusted R Squared = .062) | | | | | | | | | |
| ah. R Squared = .759 (Adjusted R Squared = .658) | | | | | | | | | |
| ai. R Squared = .911 (Adjusted R Squared = .874) | | | | | | | | | |
| aj. R Squared = .733 (Adjusted R Squared = .622) | | | | | | | | | |
| ak. R Squared = .964 (Adjusted R Squared = .949) | | | | | | | | | |
| al. R Squared = .892 (Adjusted R Squared = .847) | | | | | | | | | |
| am. R Squared = .845 (Adjusted R Squared = .781) | | | | | | | | | |
| an. R Squared = .813 (Adjusted R Squared = .735) | | | | | | | | | |
| ao. R Squared = .921 (Adjusted R Squared = .888) | | | | | | | | | |
| ap. R Squared = .927 (Adjusted R Squared = .897) | | | | | | | | | |
| aq. R Squared = .722 (Adjusted R Squared = .605) | | | | | | | | | |
| ar. R Squared = .383 (Adjusted R Squared = .125) | | | | | | | | | |
| as. R Squared = .804 (Adjusted R Squared = .722) | | | | | | | | | |
| at. R Squared = .752 (Adjusted R Squared = .649) | | | | | | | | | |
| au. R Squared = .999 (Adjusted R Squared = .999) | | | | | | | | | |
| av. R Squared = .962 (Adjusted R Squared = .947) | | | | | | | | | |
| aw. R Squared = .003 (Adjusted R Squared = -.412) | | | | | | | | | |
| ax. R Squared = .091 (Adjusted R Squared = -.288) | | | | | | | | | |
| ay. R Squared = .581 (Adjusted R Squared = .407) | | | | | | | | | |
| az. R Squared = .900 (Adjusted R Squared = .858) | | | | | | | | | |
| ba. R Squared = .810 (Adjusted R Squared = .731) | | | | | | | | | |
| bb. R Squared = .886 (Adjusted R Squared = .838) | | | | | | | | | |
| bc. Computed using alpha = .05 | | | | | | | | | |
